# A Dual‐Functional Nanoplatform Targeting the dsDNA–cGAS Axis to Mitigate Disc Degeneration and Aberrant Innervation

**DOI:** 10.1002/advs.78215

**Published:** 2026-10-12

**Authors:** Zhaojun He, Tianyu Chen, Zezheng Liu, Xinlin Huang, Ruitangbin Jiang, Yihao Liu, Baojing Xu, Yuxiang Li, Kangning Zhao, Huibo Yan, Lei Zhou, Can Liu, Qingchu Li

**Affiliations:** ^1^ Department of Orthopedics Orthopedic Hospital of Guangdong Province Academy of Orthopedics Guangdong Province The Third Affiliated Hospital of Southern Medical University Guangzhou China; ^2^ Department of Orthopaedic Surgery Guangzhou Key Laboratory of Spine Disease Prevention and Treatment The Third Affiliated Hospital Guangzhou Medical University Guangzhou China

**Keywords:** cellular senescence, disc degeneration, dsDNA–cGAS axis, nanoparticles, pathological sensory innervation

## Abstract

Pathological sensory nerve ingrowth into degenerated intervertebral discs contributes to discogenic pain, yet strategies that concurrently mitigate disc degeneration and pathological innervation remain limited. Here, we found that senescent nucleus pulposus (NP) cells accumulated and released double‐stranded DNA (dsDNA), which reinforced NP cell senescence and matrix catabolism. The accumulated dsDNA also activated cGAS–STING signaling, increased Netrin‐1 expression, and promoted neuronal migration and neurite outgrowth. Guided by these findings, we developed RU@PEI‐ZIF, a dual‐functional nanoplatform combining PEI‐mediated dsDNA scavenging with ZIF‐8‐mediated delivery of the cGAS inhibitor RU.521. RU@PEI‐ZIF displayed dsDNA‐binding capacity, cytocompatibility, and pH‐responsive RU.521 release. In senescent NP cells, RU@PEI‐ZIF reduced extracellular and cytosolic dsDNA, attenuated cGAS–STING‐associated inflammatory and neurotrophic signaling, and reduced NP cell‐mediated neuronal migration and neurite outgrowth. In an animal model of disc degeneration, intradiscal RU@PEI‐ZIF reduced disc degeneration, NP cell senescence, pathological sensory innervation, and pain hypersensitivity. These findings support RU@PEI‐ZIF as a dual‐action nanotherapeutic strategy targeting both disc degeneration and pathological innervation.

## Graphical abstract

RU@PEI‐ZIF integrates dsDNA scavenging with local cGAS inhibition to suppress the dsDNA–cGAS–STING/Netrin‐1 axis in intervertebral disc degeneration. By attenuating nucleus pulposus cell senescence, inflammatory and matrix‐catabolic responses, and pathological sensory innervation, this dual‐functional nanoplatform preserves disc integrity and reduces pain hypersensitivity, providing a mechanism‐guided therapeutic strategy for disc degeneration and discogenic pain.

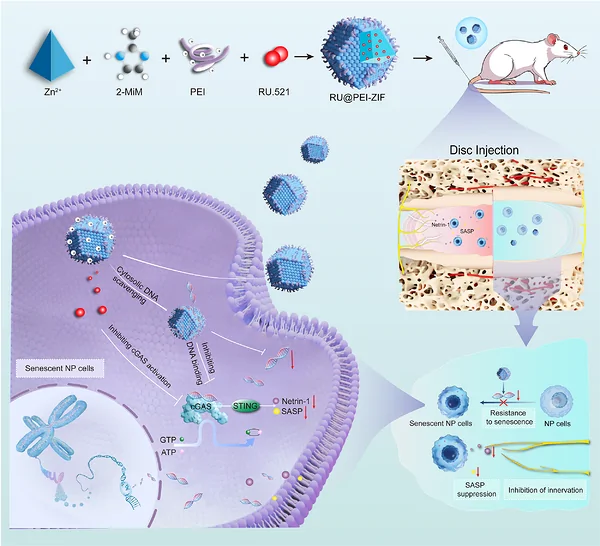

## 1 Introduction

Disc degeneration represents a key pathological factor underlying low back pain (LBP), contributing substantially to disability and socioeconomic burden [1]. This degeneration disrupts microenvironmental homeostasis, thereby facilitating aberrant sensory nerve fiber infiltration into the aneural nucleus pulposus (NP) [2, 3]. This ectopic innervation, together with neuronal sensitization, provides an anatomical basis for discogenic pain [4, 5]. However, current therapeutic options for discogenic pain, such as nonsteroidal anti‐inflammatory drugs (NSAIDs), physiotherapeutic interventions, surgical procedures, and emerging regenerative strategies, remain palliative and fail to suppress pathological nerve ingrowth [6, 7]. Inhibiting aberrant sensory innervation may provide therapeutic benefits, but the molecular mechanisms that drive pathological nerve infiltration remain poorly defined.

Accumulating evidence indicates that senescent cells release pathogenic mediators that contribute to aberrant sensory innervation in degenerative musculoskeletal disorders [8, 9]. In the degenerative intervertebral discs, senescent NP cells undergo stable cell‐cycle arrest and acquire a senescence‐associated secretory phenotype (SASP) [10, 11, 12]. Persistent DNA damage, a hallmark of cellular senescence, can lead to cytosolic double‐stranded DNA (dsDNA) accumulation and extracellular dsDNA release [13]. Cytosolic dsDNA is sensed by cyclic GMP–AMP synthase (cGAS), which subsequently activates stimulator of interferon genes (STING) signaling [14, 15]. Evidence from other injured tissues further suggests that extracellular self‐DNA can transmit senescence‐associated signals to neighboring cells [16]. Beyond inflammation, cGAS–STING signaling has also been implicated in VEGFA‐mediated angiogenesis, suggesting a broader role in tissue remodeling [17]. However, whether dsDNA released by senescent NP cells promotes senescence in other NP cells remains unclear. It is likewise unknown whether cGAS–STING activation couples senescence‐associated DNA stress to axon‐guidance cue expression and the acquisition of a pro‐innervation phenotype. Clarifying these relationships could provide a rationale for targeting dsDNA–cGAS signaling to limit pathological nerve ingrowth and alleviate discogenic pain.

Although pharmacological inhibition of cGAS–STING signaling represents a potential strategy for disc degeneration, effective drug delivery is hindered by the avascular disc and limited intradiscal retention [18, 19]. Metal–organic frameworks (MOFs), particularly the acid‐labile zeolitic imidazolate framework‐8 (ZIF‐8), offer high drug‐loading capacity and pH‐responsive release [20, 21, 22]. However, ZIF‐8 primarily serves as a delivery scaffold and lacks intrinsic capacity to scavenge accumulated dsDNA. Surface modification offers an effective strategy to overcome this limitation by introducing additional biological functions [23]. Specifically, cationic polymer modification enables nanoparticles to bind negatively charged dsDNA and thereby reduce DNA‐induced immune activation, but excessive cationic charge may cause cytotoxicity [24, 25]. Thus, integrating a DNA‐binding cationic surface with a drug‐loaded MOF core provides a synergistic strategy for dsDNA capture and controlled local inhibitor release.

In this study, we identified dsDNA–cGAS–STING signaling as a mechanistic link between NP cell senescence and the acquisition of a pro‐innervation phenotype. Guided by this mechanism, we developed RU@PEI‐ZIF, a dual‐functional nanoplatform that combines dsDNA sequestration with local cGAS inhibition (Figure 1). Across cellular and rat disc degeneration models, RU@PEI‐ZIF attenuated NP cell senescence, ECM disruption, pathological sensory innervation, and pain‐related behavior, supporting its potential as a mechanism‐guided nanotherapeutic strategy for discogenic pain.

**FIGURE 1:**
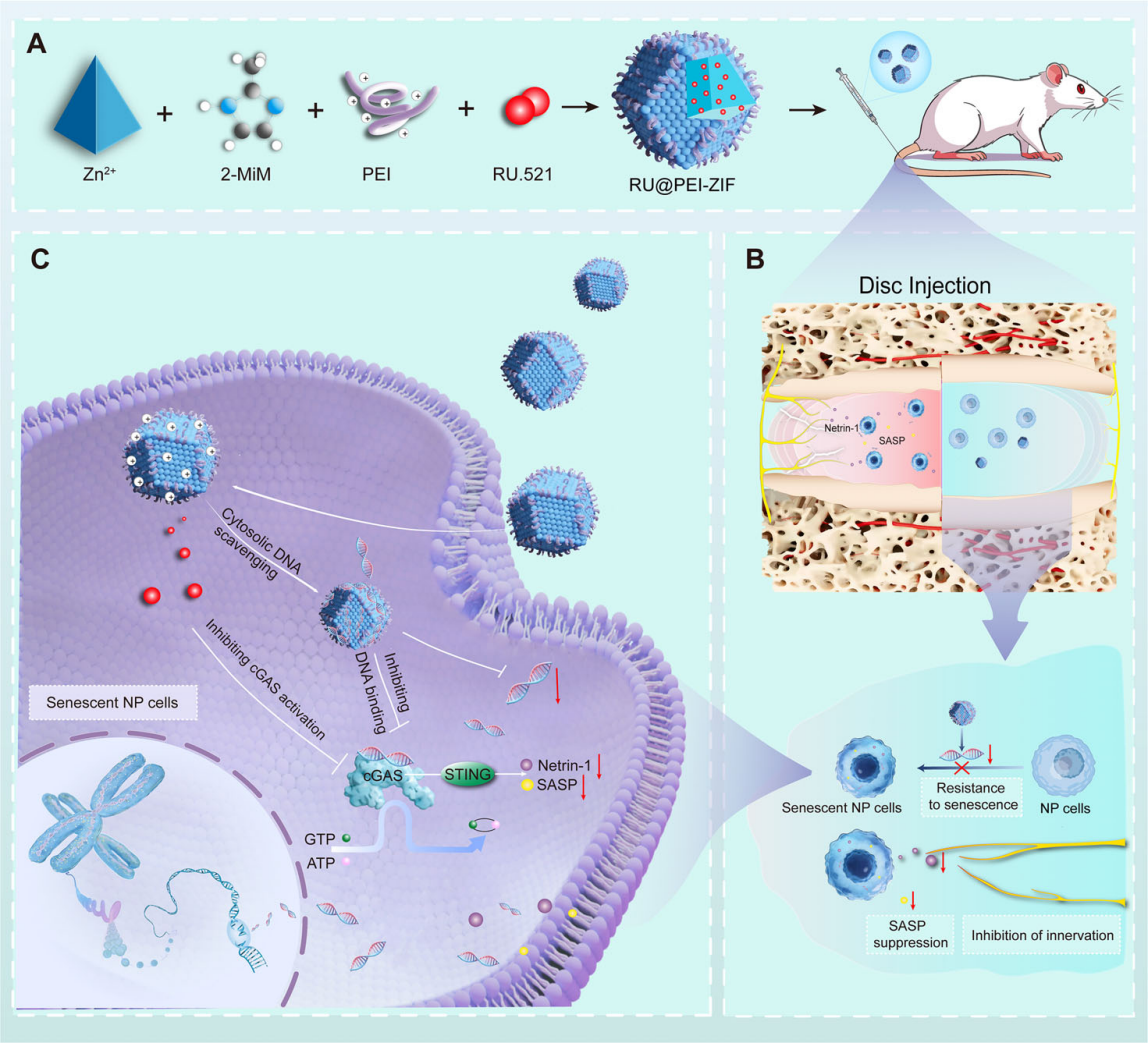
Schematic illustration of the design and function of RU@PEI‐ZIF. A) RU@PEI‐ZIF synthesis through RU.521 loading into ZIF‐8 nanoparticles and PEI coating. B) After intradiscal injection, RU@PEI‐ZIF delays disc degeneration and attenuates NP cell senescence and pathological innervation. C) Within NP cells, RU@PEI‐ZIF captures dsDNA and inhibits cGAS–STING signaling, reducing SASP factors and Netrin‐1 secretion.

## 2 Results

### 2.1 Senescent NP Cells are Associated with Aberrant Sensory Innervation and Pain Hypersensitivity

Cellular senescence is a prominent feature of intervertebral disc degeneration (IVDD) [26, 27]. To systematically characterize the senescence‐associated phenotypic alterations in NP cells and their association with pain‐related neural programs, five publicly available single‐cell RNA‐seq datasets were reanalyzed. Unsupervised clustering identified nine major cell populations in control and IVDD samples, with NP cells comprising the largest fraction (Figure 2A). Subsequent subclustering resolved five NP cell subsets: homeostatic (SR‐NPCs), adhesion‐ and lipid metabolism‐associated (Adh‐NPCs), inflammation‐responsive (IR‐NPCs), senescence‐enriched (Sen‐NPCs), and proliferative (Pro‐NPCs) populations (Figure 2B). Projection of the SenMayo scores demonstrated that senescence‐associated transcriptional programs were predominantly enriched in the Sen‐NPC subset (Figure 2C,D). The proportion of Sen‐NPCs increased from 10.0% in control samples to 20.1% in IVDD samples, whereas the proportion of SR‐NPCs decreased from 31.6% to 28.6% (Figure 2E). Gene Ontology (GO) and Kyoto Encyclopedia of Genes and Genomes (KEGG) enrichment analyses identified biological processes and pathways associated with oxidative stress, DNA damage, cellular senescence, and extracellular matrix (ECM) remodeling. NF‐κB, p53, TNF, and chemokine signaling pathways were also enriched in Sen‐NPCs (Figure 2F). We next examined whether this senescence‐enriched state was associated with neural signaling linked to aberrant sensory nerve ingrowth. Cell–cell communication analysis indicated greater predicted ligand–receptor interaction counts and signaling strength between Sen‐NPCs and neural cells in IVDD samples relative to control samples (Figure 2G).

**FIGURE 2:**
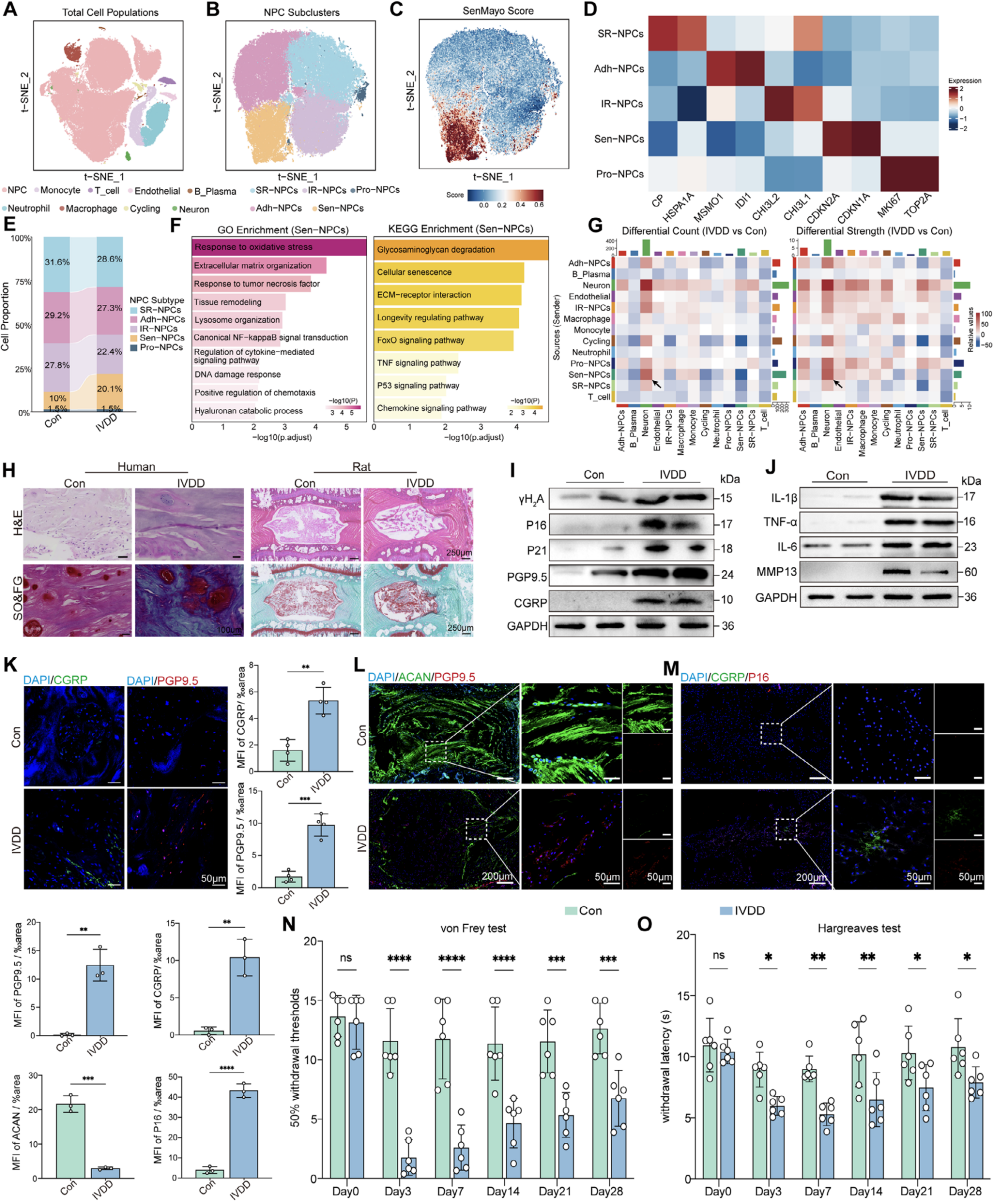
Senescent NP cells are associated with pathological sensory innervation and pain sensitization. A) t‐SNE visualization of single‐cell transcriptomes derived from human discs. B) t‐SNE map of NP cells resolving five transcriptionally distinct NP subsets. C) Projection of the SenMayo senescence score onto the NP cells t‐SNE embedding. D) Marker gene expression in each cluster by heatmap. E) Bar plots show the relative percentage of cell clusters in control and IVDD groups. F) Functional enrichment of differentially expressed genes in senescent NP cells, analyzed via GO and KEGG pathways. G) Cell–cell communication networks illustrating the total number of interactions (left) and overall signaling intensity (right) across major disc cell populations. H) Representative H&E and SO&FG staining of human and rat intervertebral disc tissues from control and IVDD groups. I) Western blot analysis of NP tissues for γH_2_A, P16, P21, PGP9.5, and CGRP. J) Western blot analysis of NP tissues for IL‐1β, TNF‐α, IL‐6, and MMP13. K) Immunofluorescence imaging and corresponding quantification of CGRP and PGP9.5 in human intervertebral disc tissues from control and IVDD groups (*n* = 4). L) Immunofluorescence imaging and corresponding quantification of aggrecan and PGP9.5 in rat intervertebral disc tissues (*n* = 3). M) Immunofluorescence imaging and corresponding quantification of P16 and CGRP in rat intervertebral disc tissues (*n* = 3). N) Mechanical sensitivity was assessed using the von Frey test, with 50% tail withdrawal thresholds measured in control and IVDD groups (*n* = 6). O) Thermal hyperalgesia was evaluated via the Hargreaves test in response to heat stimulation in control and IVDD groups (*n* = 6). ns, not significant; ^*^ *p* < 0.05, ^**^ *p* < 0.01, ^***^ *p* < 0.001, ^****^ *p* < 0.0001.

To determine whether these transcriptomic changes were recapitulated at the tissue level, we analyzed human control and degenerative disc specimens. T2‐weighted MRI showed reduced disc signal intensity and structural disruption in IVDD samples (Figure S1A). Histological examination revealed degenerative structural changes, while immunohistochemical analyses showed increased P16, P21, and MMP13 expression together with reduced aggrecan staining (Figure 2H; Figure S1B,C). We then assessed the same pathological features in a rat caudal disc needle‐puncture model. Four weeks after injury, hematoxylin and eosin (H&E) and Safranin O/Fast Green (SO&FG) staining revealed tissue disorganization and matrix depletion in punctured discs (Figure 2H). Western blot analysis revealed increased levels of the DNA damage marker γH_2_A and the senescence markers P16 and P21 in IVDD discs. The pan‐neuronal marker PGP9.5 and the nociceptive neuropeptide CGRP were also increased (Figure 2I; Figure S2A). Similarly, the pro‐inflammatory cytokines IL‐1β, TNF‐α, and IL‐6, together with the matrix‐degrading enzyme MMP13, were increased in IVDD discs (Figure 2J; Figure S2B). These molecular changes were corroborated by immunofluorescence, which showed increased γH_2_A, IL‐1β, TNF‐α, and MMP13 signals in IVDD tissues (Figure S3A).

Having established a senescence‐associated inflammatory phenotype in degenerative discs, we next examined pathological sensory innervation and vascular ingrowth. In human degenerative disc specimens, immunofluorescence showed increased PGP9.5^+^ nerve fibers and CGRP^+^ peptidergic nociceptive fibers relative to controls (Figure 2K). Rat IVDD discs displayed a similar increase in PGP9.5^+^ fibers, accompanied by marked aggrecan loss (Figure 2L). These fibers were concentrated at the annulus fibrosus‐NP interface, although some extended into the NP. CD31^+^ vascular structures were likewise more abundant in rat IVDD discs (Figure S3B). Moreover, regions with prominent P16 staining were spatially associated with increased CGRP^+^ fiber accumulation (Figure 2M). Longitudinal behavioral assessments over a 4‐week period showed that rats with disc degeneration developed persistent thermal hyperalgesia and mechanical hypersensitivity. Specifically, compared with the control group, the IVDD group exhibited a progressive and sustained reduction in the 50% withdrawal threshold at each post‐injury time point (Figure 2N). In parallel, thermal withdrawal latency was also reduced at each post‐injury time point (Figure 2O). Together, these data support a framework in which disc degeneration is associated with expansion of senescent NP cells, activation of DNA damage responses, inflammatory signaling, and matrix catabolism. The molecular and spatial association between NP cell senescence and sensory nerve infiltration suggests that senescent NP cells may contribute to a degenerative microenvironment permissive to pathological neurovascular ingrowth and pain hypersensitivity.

### 2.2 Senescent NP Cells Accumulate and Release dsDNA that Reinforces Senescence and Pro‐Innervation Activity

To examine how NP cell senescence might support aberrant sensory nerve ingrowth, we compared early‐passage rat NP cells (Control) with replicatively senescent passage 8 cells (SEN) (Figure 3A). Bulk RNA sequencing (RNA‐seq) revealed broad transcriptomic differences between replicatively senescent P8 and early‐passage NP cells. Genes involved in cell‐cycle regulation and DNA repair were markedly dysregulated. For example, the cell‐cycle regulators *Cdkn1a* and *Cdkn2a* were upregulated, whereas the DNA repair genes *Mgmt* and *Nhej1* were downregulated (Figure 3B). Pathway analysis showed enrichment of cellular senescence, DNA damage, p53 and TNF signaling, and ECM remodeling among the upregulated genes. Downregulated genes were associated mainly with DNA repair, particularly double‐strand break repair (Figure 3C). GSEA identified enrichment of the DNA damage response and DNA damage sensor activity in P8 cells (Figure 3D,E). Immunofluorescence showed greater γH_2_A signals and cytosolic dsDNA accumulation in P8 cells than in early‐passage controls (Figure 3F). NP cells were exposed to H_2_O_2_ to establish a senescent model of the IVDD oxidative microenvironment. This independent stress model likewise showed increased γH_2_A signals and cytosolic dsDNA accumulation (Figure 3F). Quantification of conditioned‐media dsDNA further showed increased extracellular dsDNA levels in both replicatively senescent and H_2_O_2_‐treated cultures (Figure 3G). RNA‐seq followed by pathway analysis of H_2_O_2_‐treated NP cells revealed enrichment of oxidative stress‐, DNA damage‐, DNA repair‐, and cellular senescence‐related pathways (Figure S4A,B). GSEA showed enrichment of double‐strand break repair and positive regulation of neuron projection development (Figure S4C,D).

**FIGURE 3:**
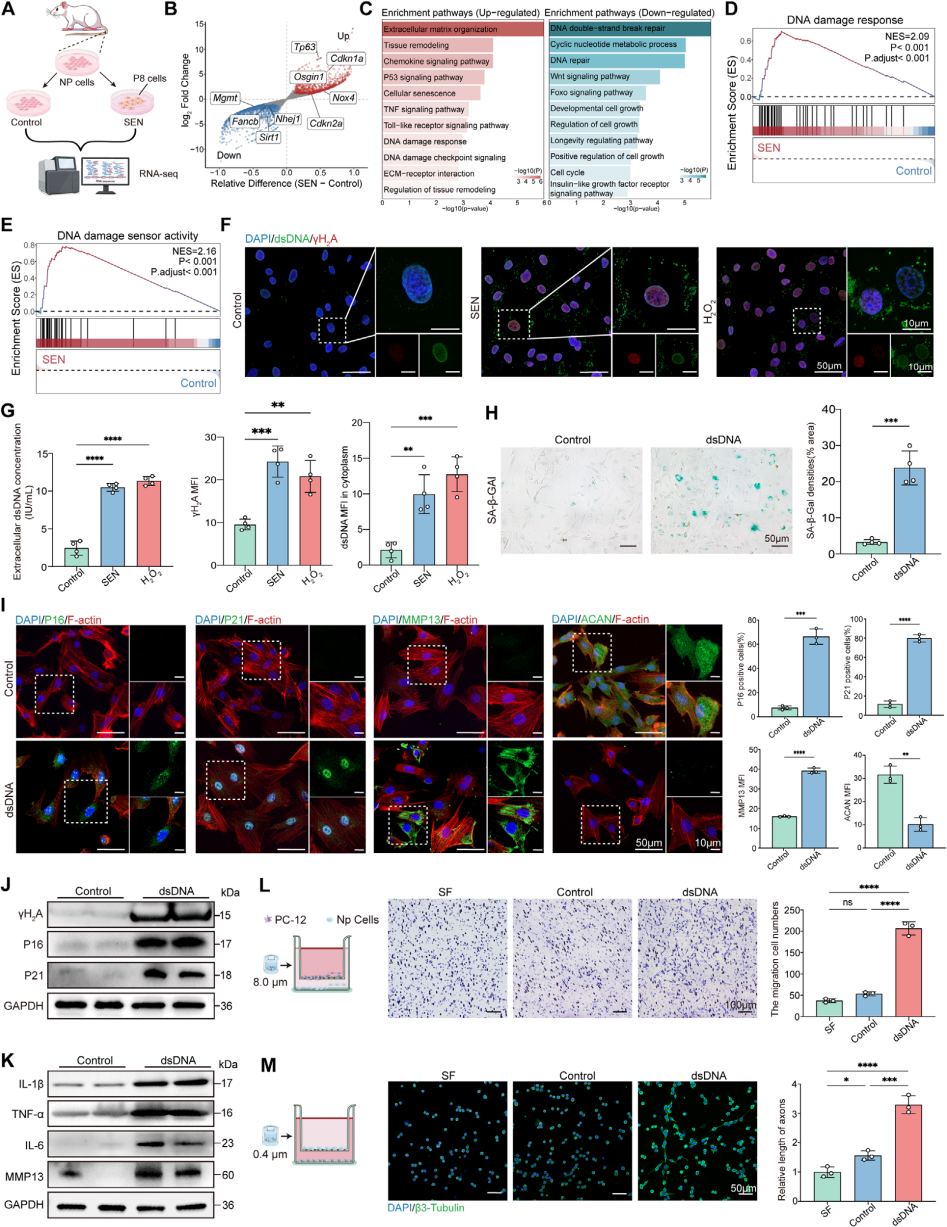
Intra‐ and extracellular dsDNA drive the senescence–innervation axis in NP cells. A) Overview of the RNA‐seq experimental workflow comparing low‐passage NP cells (Control) and replicatively senescent P8 NP cells (SEN). B) Differential gene expression between Control and SEN is displayed in a volcano plot. C) Enrichment analysis of upregulated (left) and downregulated (right) pathways between Control and SEN NP cells. D, E) GSEA showing enrichment of the DNA damage response (D) and DNA damage sensor activity (E) in SEN NP cells relative to control NP cells. F) Immunofluorescence imaging and corresponding quantification of γH_2_A and dsDNA in Control, SEN, and H_2_O_2_‐treated NP cells (*n* = 4). G) ELISA quantification of extracellular dsDNA in conditioned media from Control, SEN, and H_2_O_2_‐treated NP cells (*n* = 4). H) SA‐β‐Gal staining in Control and dsDNA‐treated NP cells, with quantitative assessment of positive cells (*n* = 4). I) Immunofluorescence imaging and corresponding quantification of P16, P21, MMP13, and aggrecan in Control and dsDNA‐treated NP cells (*n* = 3). J) Western blot analysis of γH_2_A, P16, and P21 in Control and dsDNA‐treated NP cells. K) Western blot analysis of IL‐1β, TNF‐α, IL‐6, and MMP13 in Control and dsDNA‐treated NP cells. L) Representative Transwell images and quantification of PC12 cell migration across experimental conditions (*n* = 3). M) Representative β3‐tubulin immunofluorescence images showing PC12 neurite outgrowth, with corresponding quantification of relative neurite length (*n* = 3). SF, serum‐free medium; Control, low‐passage NP cells; SEN, replicatively senescent P8 NP cells. ns, not significant; ^*^ *p* < 0.05, ^**^ *p* < 0.01, ^***^ *p* < 0.001, ^****^ *p* < 0.0001.

Because senescent NP cells accumulated and released dsDNA, we next tested whether extracellular dsDNA was sufficient to induce a senescence‐associated phenotype. Exposure of early‐passage NP cells to exogenous dsDNA increased the proportion of SA‐β‐Gal‐positive cells (Figure 3H). Immunofluorescence further showed increased P16, P21, and MMP13 signals together with reduced aggrecan expression (Figure 3I). Consistent with these findings, Western blotting and densitometric analysis demonstrated increased γH_2_A, P16, and P21 levels after dsDNA treatment (Figure 3J; Figure S5A), together with elevated IL‐1β, TNF‐α, IL‐6, and MMP13 levels (Figure 3K; Figure S5B). To test the functional contribution of released dsDNA, DNase I was applied to replicatively senescent and H_2_O_2_‐treated NP‐cell cultures to degrade extracellular dsDNA in the culture medium [17, 28]. DNase I reduced SA‐β‐Gal positivity in both models (Figure S6A). It also lowered P16, P21, and MMP13 signals while partially restoring aggrecan (Figure S6B). These reciprocal perturbations supported a functional contribution of extracellular dsDNA to the senescent and catabolic NP‐cell phenotype.

Finally, we asked whether dsDNA‐treated NP cells altered neuronal migration and neurite growth. In Transwell assays, dsDNA‐treated NP cells elicited greater PC12 migration than control NP cells or serum‐free medium alone (Figure 3L). Non‐contact co‐culture with dsDNA‐treated NP cells increased β3‐tubulin‐positive neurite extension in PC12 cells compared with control NP cells (Figure 3M). Taken together, these findings show that senescent NP cells accumulate and release dsDNA, and that extracellular dsDNA reinforces the senescent and catabolic NP‐cell phenotype while promoting NP cell‐mediated neuronal migration and neurite outgrowth.

### 2.3 DsDNA Promotes Netrin‐1‐Associated Pro‐Innervation Activity through cGAS–STING Signaling

Having established that extracellular dsDNA was sufficient to induce a senescence‐associated, pro‐innervation phenotype in NP cells, we investigated the signaling program linking DNA stress to neuronal responses. RNA‐seq of dsDNA‐stimulated NP cells revealed widespread transcriptional changes (Figure 4A). GO analysis linked these transcriptional changes to cellular senescence, cell‐cycle regulation, tissue and ECM remodeling, DNA damage responses, and DNA‐damage checkpoint signaling (Figure 4B). GSEA showed enrichment of replicative senescence and cGAS–STING signaling, identifying cytosolic DNA sensing as a candidate link between dsDNA exposure and the altered NP‐cell secretory state (Figure 4C,D). dsDNA also increased inflammatory and SASP transcripts, including *Il1b*, *Il6*, *Cxcl1*, *Ccl2*, and *Mmp3* (Figure 4E). The positive regulation of neuron migration gene set was enriched in dsDNA‐treated NP cells, accompanied by increased expression of *Ntn1*, *Ntrk2*, *Nrp1*, *Nrp2*, *Sema5a*, and *Slit2* (Figure 4F,G). *Ntn1* was among the most strongly induced genes. Given that *Ntn1* encodes the neurotrophic axon‐guidance cue Netrin‐1, we next examined its role in the dsDNA‐induced pro‐innervation phenotype [29, 30]. To determine whether this cGAS–Netrin‐1 expression pattern was also evident in human disc degeneration, we performed immunohistochemical analysis of human disc specimens. cGAS and Netrin‐1 expression were higher in IVDD tissues than in non‐degenerative controls (Figure S1D). Consistent with the dsDNA‐stimulation model, H_2_O_2_‐treated NP cells also showed increased cGAS, STING, and Netrin‐1 signals relative to controls (Figure S4E).

**FIGURE 4:**
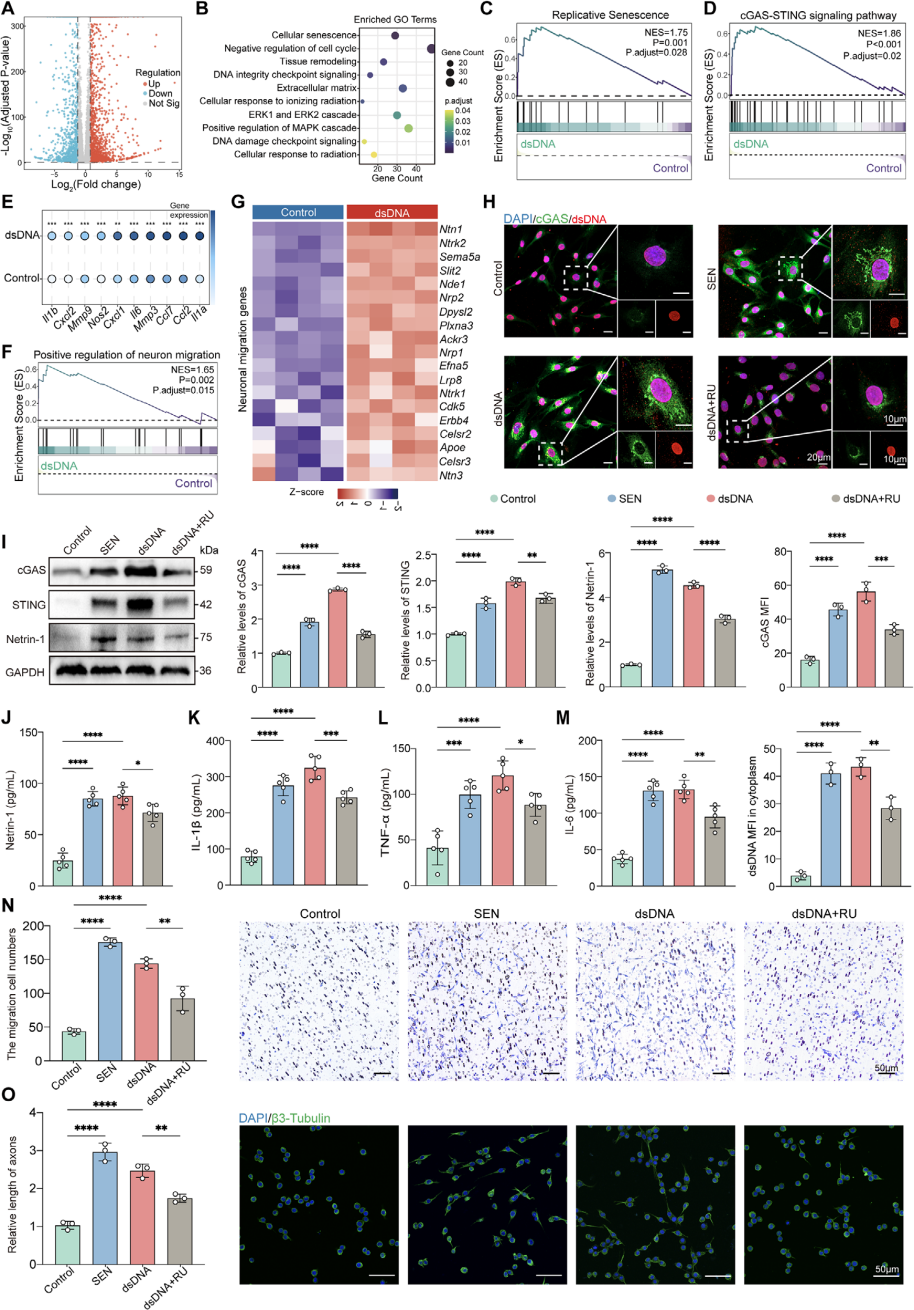
Activation of cGAS–STING signaling in NP cells induces Netrin‐1 expression to promote neurite growth. A, B) Volcano plot (A) and GO enrichment analysis (B) displaying differentially expressed genes in dsDNA‐treated NP cells relative to control NP cells. C, D) GSEA showing enrichment of replicative senescence (C) and the cGAS–STING signaling pathway (D) in dsDNA‐treated NP cells relative to control NP cells. E) Dot plot showing the expression of representative inflammatory and SASP‐associated genes in Control and dsDNA‐treated NP cells. F) GSEA showing enrichment of positive regulation of neuron migration in dsDNA‐treated NP cells relative to control NP cells. G) Heatmap of representative neuron migration‐associated genes in Control and dsDNA‐treated NP cells. H) Immunofluorescence imaging and corresponding quantification of dsDNA and cGAS across the indicated groups (*n* = 3). I) Western blot analysis and quantification of cGAS, STING, and Netrin‐1 in different groups (*n* = 3). J–M) ELISA quantification of secreted Netrin‐1 (J) and SASP‐associated cytokines, including IL‐1β (K), TNF‐α (L), and IL‐6 (M) in conditioned media from the indicated groups (*n* = 5). N) Representative Transwell images and quantification of PC12 cell migration assays in different groups (*n* = 3). O) Representative β3‐tubulin immunofluorescence images showing PC12 neurite outgrowth, with corresponding quantification of relative neurite length (*n* = 3). dsDNA + RU, dsDNA‐stimulated NP cells subsequently treated with the cGAS inhibitor RU.521. ^*^ *p* < 0.05, ^**^ *p* < 0.01, ^***^ *p* < 0.001, ^****^ *p* < 0.0001.

To genetically validate the RNA‐seq findings, cGAS was knocked down using siRNA. GFP signals confirmed successful transfection, whereas Western blotting analysis showed that si‐cGAS‐1 and si‐cGAS‐2 achieved the greatest cGAS knockdown (Figure S7A,B). Under dsDNA stimulation, STING, Netrin‐1, IL‐1β, and TNF‐α were elevated in control‐transfected NP cells, and these increases were attenuated following cGAS knockdown (Figure S7C). In Transwell assays, dsDNA‐stimulated control‐transfected NP cells promoted PC12 migration, an effect attenuated by cGAS knockdown. Likewise, cGAS knockdown attenuated β3‐tubulin‐positive neurite extension by PC12 cells during non‐contact co‐culture (Figure S7D).

Although siRNA‐mediated cGAS knockdown provided genetic validation, its therapeutic translation is constrained by delivery and safety concerns [31]. Small‐molecule inhibitors, which enable dose‐titratable pharmacological targeting, have therefore attracted increasing interest. Accordingly, RU.521 (RU), a small‐molecule cGAS inhibitor, was selected for pharmacological evaluation [32]. Replicatively senescent and dsDNA‐treated NP cells showed stronger cytosolic dsDNA and cGAS signals than control cells, whereas RU.521 reduced both signals after dsDNA stimulation (Figure 4H). Western blotting with densitometric analysis showed increased cGAS, STING, and Netrin‐1 levels in replicatively senescent and dsDNA‐treated NP cells, whereas RU.521 attenuated the increases induced by dsDNA stimulation (Figure 4I). Conditioned media from senescent and dsDNA‐treated cultures contained more Netrin‐1, IL‐1β, TNF‐α, and IL‐6 than control conditioned media, and RU.521 reduced each dsDNA‐induced increase (Figure 4J–M). Functionally, replicatively senescent and dsDNA‐treated NP cells elicited greater PC12 migration in Transwell assays, whereas RU.521 attenuated this response (Figure 4N). In non‐contact co‐culture, RU.521 also reduced β3‐tubulin‐positive neurite extension induced by these NP cells (Figure 4O).

We then determined whether Netrin‐1 was a functional downstream mediator. GFP signals indicated successful transfection, and densitometric Western blot analysis confirmed Netrin‐1 knockdown by all three siRNAs (Figure S8A,B). Silencing Netrin‐1 in senescent NP cells diminished their ability to recruit PC12 cells across Transwell inserts and to support β3‐tubulin‐positive neurite outgrowth in non‐contact co‐culture (Figure S8C,D). In vivo, rat discs received intradiscal AAV‐NTN1 shRNA or AAV‐null 1 week before needle puncture. Four weeks after injury, AAV‐null‐treated degenerative discs contained abundant CGRP^+^ sensory fibers and PGP9.5^+^ neuronal fibers, whereas AAV‐mediated Netrin‐1 knockdown reduced both signals (Figure S8E). Together, these genetic and pharmacological findings support a dsDNA–cGAS–STING–Netrin‐1 axis that promotes the pro‐innervation phenotype of senescent NP cells and provides a rationale for cGAS inhibition in IVDD.

### 2.4 Synthesis and Physicochemical Characterization of RU@PEI‐ZIF Nanoplatform

To achieve the dual objectives of RU.521 delivery and dsDNA capture, we engineered a dual‐functional nanoplatform based on a ZIF‐8 architecture. Specifically, RU.521 was incorporated during PEI‐templated ZIF‐8 assembly to generate RU@PEI‐ZIF. Scanning electron microscopy (SEM) and transmission electron microscopy (TEM) showed that RU@ZIF and RU@PEI‐ZIF retained well‐defined polyhedral morphologies without obvious structural collapse (Figure 5A). Annular dark‐field scanning transmission electron microscopy (ADF‐STEM) elemental mapping of RU@PEI‐ZIF showed C, N, O, and Zn signals throughout the nanoparticles. A Cl signal was also detectable over the particle regions, consistent with the incorporation of Cl‐containing RU.521 (Figure 5B). Dynamic light scattering (DLS) revealed a slight increase in hydrodynamic diameter after PEI modification, while the size distribution remained unimodal, indicating that PEI incorporation did not cause obvious particle aggregation (Figure 5C). The zeta potential increased from approximately 3 mV for RU@ZIF to approximately 19 and 16 mV for PEI‐ZIF and RU@PEI‐ZIF, respectively (Figure 5D). This positive shift was consistent with the introduction of cationic PEI at the nanoparticle interface.

**FIGURE 5:**
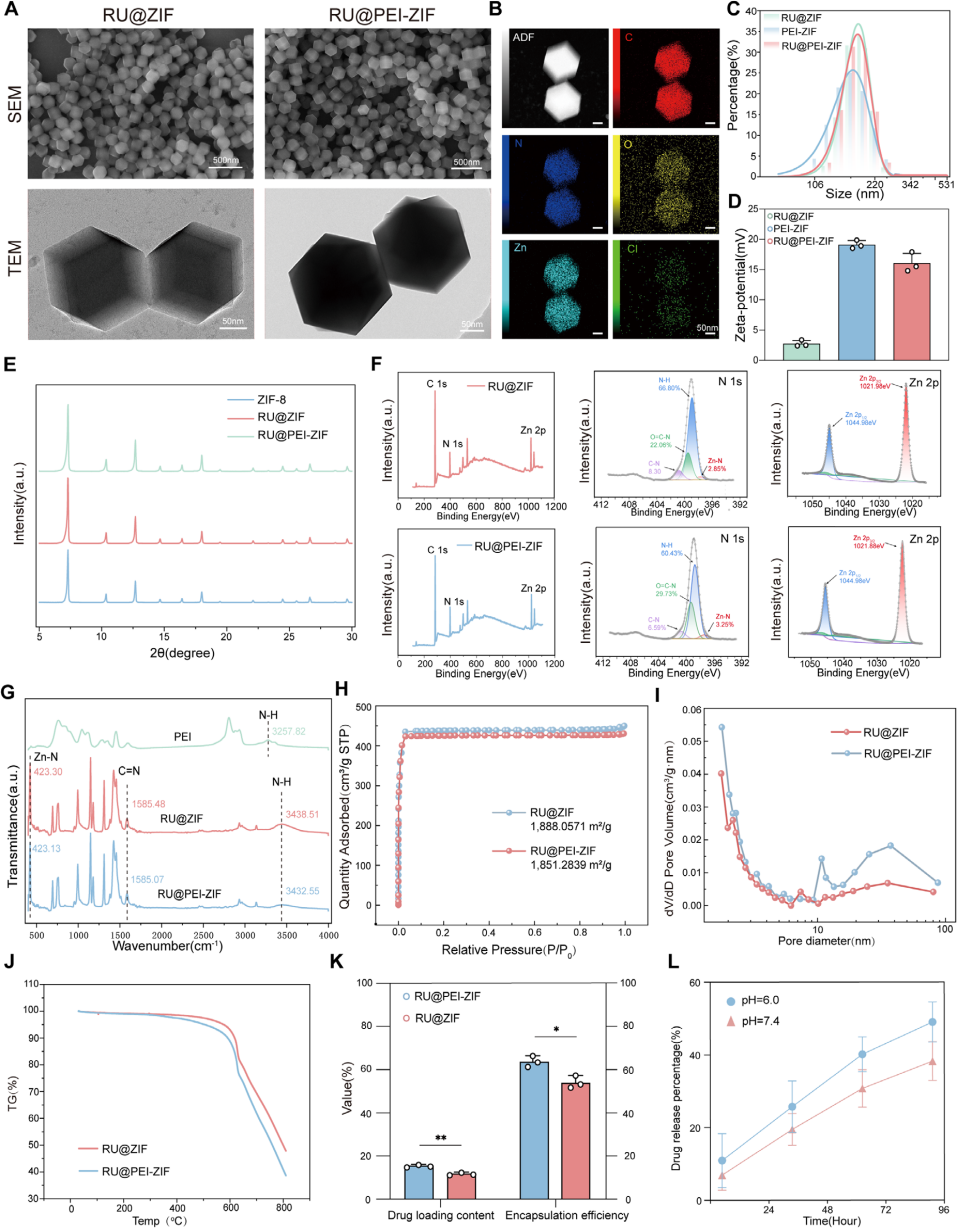
Synthesis and characterization of nanoparticles. A) Representative SEM and TEM images of RU@ZIF and RU@PEI‐ZIF, showing that polyhedral morphology is preserved after PEI functionalization. B) Elemental mapping images of RU@PEI‐ZIF, showing the distributions of C, N, O, Zn, and Cl in RU@PEI‐ZIF. C) Hydrodynamic size distributions of RU@ZIF, PEI‐ZIF, and RU@PEI‐ZIF measured by DLS. D) Zeta potentials of RU@ZIF, PEI‐ZIF, and RU@PEI‐ZIF (*n* = 3). E) XRD patterns of ZIF‐8, RU@ZIF and RU@PEI‐ZIF. F) XPS survey spectra and high‐resolution N 1s and Zn 2p spectra of RU@ZIF and RU@PEI‐ZIF. G) FT‐IR spectra of PEI, RU@ZIF, and RU@PEI‐ZIF, showing the Zn─N and C═N vibrations of the ZIF framework and the overlapping N‐H stretching region. H) N_2_ adsorption–desorption isotherms (BET) of RU@ZIF and RU@PEI‐ZIF with corresponding specific surface areas. I) Pore size distributions derived from adsorption measurements. J) Thermogravimetric analysis (TGA) profiles of RU@ZIF and RU@PEI‐ZIF. K) RU.521 drug‐loading content and encapsulation efficiency of RU@PEI‐ZIF and RU@ZIF (*n* = 3). L) pH‐dependent cumulative RU.521 release profiles of RU@PEI‐ZIF at pH 6.0 and pH 7.4 over 96 h (*n* = 3). ^*^ *p* < 0.05, ^**^ *p* < 0.01.

Next, we evaluated the crystalline integrity of the modified framework. The X‐ray diffraction (XRD) patterns of ZIF‐8, RU@ZIF, and RU@PEI‐ZIF displayed the same characteristic reflections (Figure 5E). No obvious additional reflections or peak shifts were observed after RU.521 loading and PEI incorporation. These findings indicated that both modifications preserved the crystalline structure of the ZIF‐8 framework. XPS survey spectra detected C, N, O, and Zn in both RU@ZIF and RU@PEI‐ZIF (Figure 5F). After charge referencing of the C 1s C─C/C─H signal to 284.8 eV, the Zn 2p_3_/_2_ and Zn 2p_1_/_2_ peaks were located at 1021.98 and 1044.98 eV for RU@ZIF and at 1021.88 and 1044.98 eV for RU@PEI‐ZIF, respectively. The corresponding spin–orbit separations were approximately 23.0 eV, consistent with Zn(II)‐containing ZIF‐8. Notably, PEI incorporation produced no meaningful shift in the Zn 2p binding energies. N 1s deconvolution showed that the component near 398.9 eV decreased from 66.80% to 60.43%, whereas the component near 399.4 eV increased from 22.06% to 29.73%. Given the overlapping contributions of PEI amines and ZIF‐associated nitrogen in this region, the increased 399.4 eV component was consistent with an increased contribution from PEI‐derived amine nitrogen. Together with the positive shift in zeta potential, the N 1s redistribution supported PEI incorporation, whereas preservation of the ZIF framework was independently supported by the XRD results.

FT‐IR spectra of RU@ZIF and RU@PEI‐ZIF were further compared with the spectrum of pure PEI (Figure 5G). RU@ZIF and RU@PEI‐ZIF retained the Zn‐N vibrations at 423.30 and 423.13 cm^−1^, respectively. Their imidazolate‐ring vibrations were similarly maintained at 1585.48 and 1585.07 cm^−1^, respectively. The nearly unchanged positions of these framework‐associated bands supported preservation of the ZIF structure. In the N─H region, the broad band shifted slightly from 3438.51 cm^−1^ in RU@ZIF to 3432.55 cm^−1^ in RU@PEI‐ZIF, toward the lower‐wavenumber N─H band of pure PEI. Thus, PEI functionalization was accompanied by a modest shift in the N‐H band. Because a similar broad N─H band was already present in RU@ZIF, this shift was considered a PEI‐associated spectral change rather than independent confirmation of PEI incorporation. Nitrogen adsorption‐desorption analysis yielded BET surface areas of 1888.06 and 1851.28 m^2^ g^−1^ for RU@ZIF and RU@PEI‐ZIF, respectively (Figure 5H). The limited decrease indicated that PEI incorporation caused only a modest loss of accessible surface area while retaining the highly porous ZIF architecture. The principal small‐pore features were maintained, although changes occurred within the larger‐pore and interparticle region (Figure 5I). This redistribution was consistent with changes in surface coverage and interparticle packing rather than collapse of the porous framework. Thermogravimetric analysis showed greater high‐temperature mass loss for RU@PEI‐ZIF than for RU@ZIF (Figure 5J). At 810°C, the residual masses were approximately 38.6% and 47.9%, respectively. The additional mass loss was consistent with the greater organic fraction introduced by PEI. Collectively, the zeta‐potential, N 1s, and TGA results supported PEI incorporation, while FT‐IR provided supportive but non‐specific evidence of PEI incorporation. The XRD, Zn 2p, FT‐IR, and BET results demonstrated preservation of the crystalline, coordination, vibrational, and porous features of the ZIF framework.

Finally, the drug‐loading and release properties of RU.521 were evaluated. RU@PEI‐ZIF achieved a drug‐loading content of 16.03% and an encapsulation efficiency of 66.21%. In comparison, RU@ZIF showed a drug‐loading content of 12.20% and an encapsulation efficiency of 56.07% (Figure 5K). Cumulative RU.521 release increased over 96 h and remained higher at pH 6.0 than at pH 7.4. At 96 h, approximately 49% of RU.521 was released at pH 6.0, compared with approximately 38% at pH 7.4 (Figure 5L), consistent with acid‐responsive dissociation of the ZIF framework. Together, these results established RU@PEI‐ZIF as a PEI‐functionalized, drug‐loaded ZIF platform that retained its crystalline and porous architecture while providing a cationic surface, improved RU.521 loading, and pH‐responsive release.

### 2.5 RU@PEI‐ZIF Reduces the dsDNA Burden and Attenuates NP Cell Senescence

Before testing their functional effects, we examined cellular uptake and cytocompatibility of the nanoplatforms. Confocal imaging of Cy5.5‐labeled PEI‐ZIF, RU@ZIF, and RU@PEI‐ZIF showed predominantly cytoplasmic fluorescence in NP cells after 12 h (Figure S9A). Given the potential dose‐dependent cytotoxicity associated with highly cationic materials, cytocompatibility was evaluated across a range of concentrations. CCK‐8 assays demonstrated that PEI‐ZIF, RU@ZIF, and RU@PEI‐ZIF had negligible effects on cell viability, with cells remaining highly viable at concentrations reaching 80 µg mL^−1^ (Figure S9B). A concentration of 10 µg mL^−1^ was selected for subsequent experiments. At this concentration, phalloidin staining showed intact F‐actin organization and preserved cell spreading. Calcein‐AM/PI staining likewise detected little cell death after exposure to each formulation (Figure S9C,D).

Having established a cytocompatible working concentration, we tested whether PEI functionalization enhanced dsDNA capture. In a cell‐free assay, dsDNA adsorption by RU@PEI‐ZIF increased with DNA input and remained higher than that of RU@ZIF at every concentration examined (Figure 6A). This concentration‐dependent separation was consistent with the cationic surface introduced by the PEI functionalization. We next examined whether the enhanced cell‐free dsDNA binding was associated with reduced extracellular dsDNA levels in senescent NP‐cell cultures. ELISA quantification of conditioned media showed that RU@PEI‐ZIF elicited the most pronounced decrease in extracellular dsDNA levels, outperforming PEI‐ZIF and RU@ZIF (Figure 6B). Consistently, immunofluorescence analysis showed that all three formulations attenuated cytosolic dsDNA accumulation and γH_2_A signaling in senescent NP cells, with RU@PEI‐ZIF exhibiting the strongest effect (Figure 6C). Thus, RU@PEI‐ZIF reduced the dsDNA burden in both extracellular and cytosolic compartments.

**FIGURE 6:**
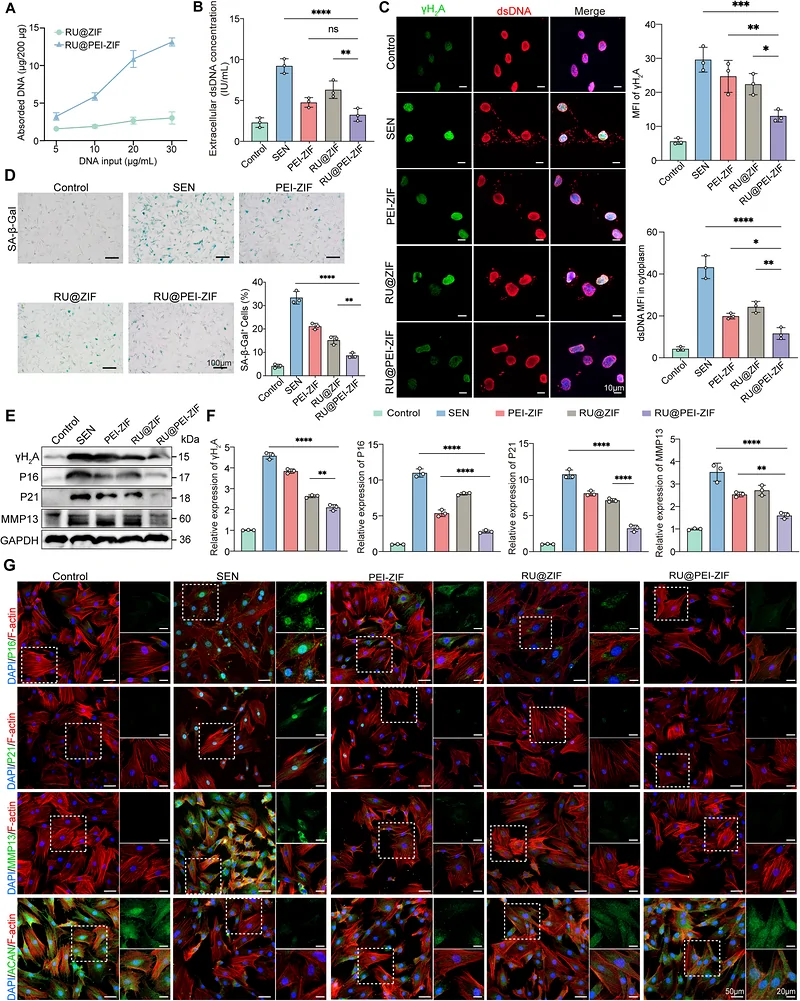
RU@PEI‐ZIF scavenges dsDNA to attenuate senescence in NP cells. A) DNA binding capacity of RU@ZIF and RU@PEI‐ZIF (*n* = 3). B) ELISA quantification of extracellular dsDNA in conditioned media across the indicated groups (*n* = 3). C) Immunofluorescence imaging and corresponding quantification of γH_2_A and dsDNA, showing accumulation of cytosolic dsDNA in senescent NP cells and its reduction following RU@PEI‐ZIF treatment (*n* = 3). D) SA‐β‐Gal staining across the indicated groups, with quantitative assessment of SA‐β‐Gal‐positive cells (*n* = 3). E, F) Western blot analysis and quantification of γH_2_A, P16, P21, and MMP13 across the indicated groups (*n* = 3). G) Immunofluorescence imaging of P16, P21, MMP13, and aggrecan across the indicated groups. ns, not significant; ^*^ *p* < 0.05, ^**^ *p* < 0.01, ^***^ *p* < 0.001, ^****^ *p* < 0.0001.

We further examined whether efficient dsDNA reduction was associated with attenuation of senescence‐associated damage and matrix catabolism. SA‐β‐Gal staining showed fewer senescent cells after nanoparticle treatment, with the lowest proportion in the RU@PEI‐ZIF group (Figure 6D). These observations were further corroborated by Western blotting and confocal immunofluorescence analyses. Western blotting showed lower γH_2_A, P16, P21, and MMP13 levels after RU@PEI‐ZIF treatment than in untreated senescent NP cells (Figure 6E,F). Immunofluorescence supported these findings, showing reduced P16, P21, and MMP13 signals together with increased aggrecan signals after RU@PEI‐ZIF treatment (Figure 6G). Taken together, RU@PEI‐ZIF treatment reduced the dsDNA burden and was accompanied by attenuation of DNA damage, cellular senescence, and matrix catabolism in NP cells.

### 2.6 RU@PEI‐ZIF Suppresses cGAS–STING to Inhibit Netrin‐1‐mediated Sensory Neurite Growth

Having established that RU@PEI‐ZIF reduced dsDNA burden and attenuated senescence‐associated damage, we next examined its effects on the cGAS–STING–Netrin‐1 axis that links cytosolic DNA sensing to aberrant innervation. Immunofluorescence revealed elevated cGAS and STING signals in senescent NP cells. PEI‐ZIF and RU@ZIF each produced only partial attenuation, whereas RU@PEI‐ZIF yielded the most pronounced suppression (Figure 7A,B). Western blotting showed reductions in cGAS, STING, Netrin‐1, IL‐1β, TNF‐α, and IL‐6 after nanoparticle treatment, with the greatest reductions observed in the RU@PEI‐ZIF group (Figure 7C; Figure S10A). ELISA further demonstrated lower release of Netrin‐1, IL‐1β, TNF‐α, and IL‐6 (Figure 7D–G). Immunofluorescence of Netrin‐1, IL‐1β, and TNF‐α showed the same pattern (Figure 7H; Figure S11A,B). Together, these results show that RU@PEI‐ZIF attenuates cGAS–STING signaling and reduces the inflammatory and neurotrophic secretory phenotype of senescent NP cells. We next assessed the functional consequences of this secretome remodeling on neuronal responses. In the Transwell co‐culture system, senescent NP cells markedly promoted PC12 chemotactic migration and β3‐tubulin‐positive neurite outgrowth. Nanoparticle treatment attenuated both responses, with RU@PEI‐ZIF producing the greatest reduction in PC12 migration and neurite outgrowth among the tested formulations (Figure 7I,J). These findings indicate that RU@PEI‐ZIF attenuates the pro‐innervation activity of senescent NP cells.

**FIGURE 7:**
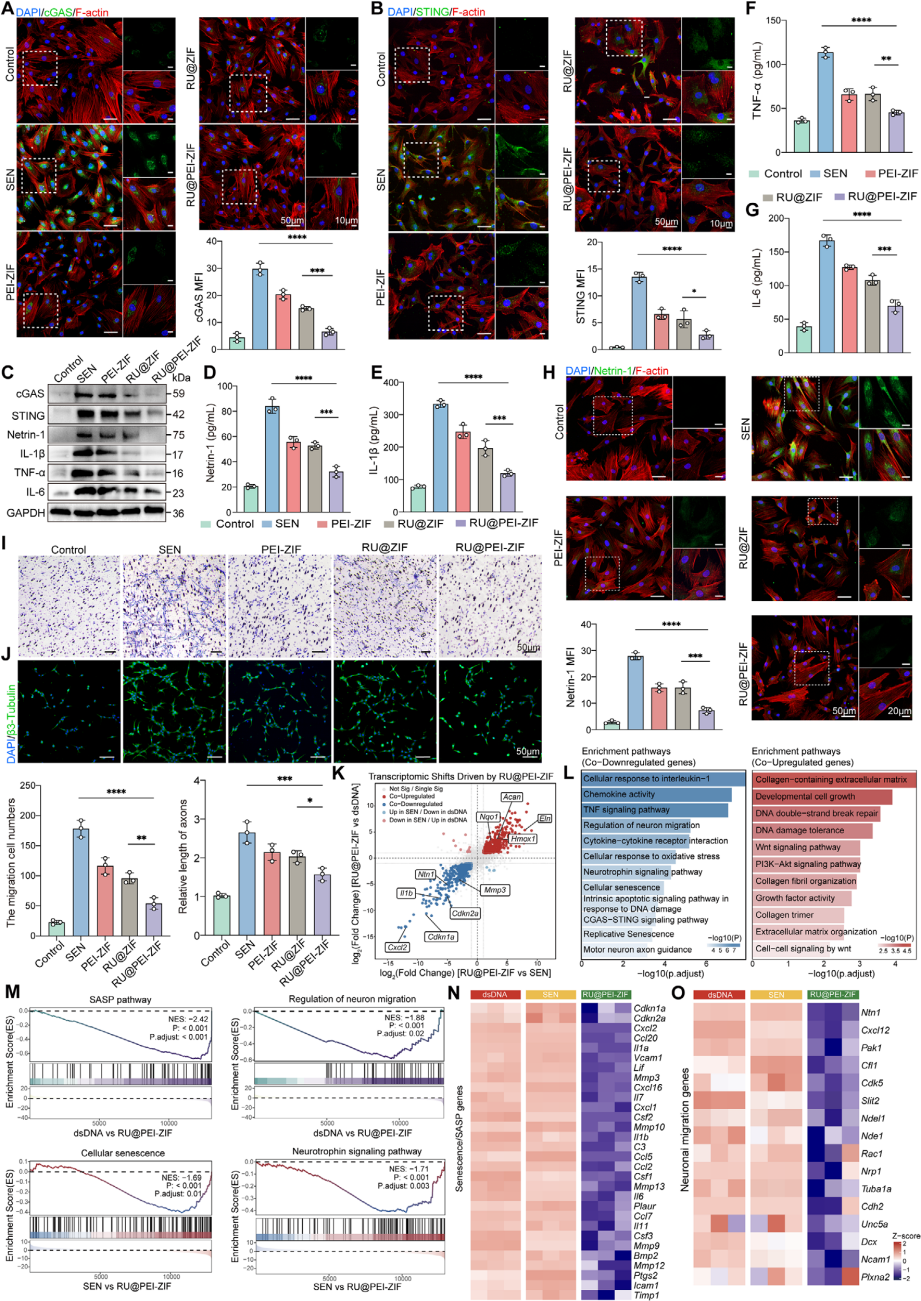
RU@PEI‐ZIF attenuates senescence‐driven pro‐innervation responses by suppressing cGAS–STING signaling. A) Immunofluorescence imaging and corresponding quantification of cGAS across the indicated groups (*n* = 3). B) Immunofluorescence imaging and corresponding quantification of STING across the indicated groups (*n* = 3). C) Western blot analysis of cGAS, STING, Netrin‐1, IL‐1β, TNF‐α, and IL‐6 across the indicated groups. D–G) ELISA quantification of secreted Netrin‐1 (D), IL‐1β (E), TNF‐α (F), and IL‐6 (G) in conditioned media from the indicated NP cell groups (*n* = 3). H) Immunofluorescence imaging and corresponding quantification of Netrin‐1 across the indicated groups (*n* = 3). I) Representative images and quantification of Transwell migration assays showing PC12 cell migration in response to conditioned media from the indicated NP cell groups (*n* = 3). J) Representative β3‐tubulin immunofluorescence images showing PC12 neurite outgrowth in response to conditioned media from the indicated NP cell groups, with corresponding quantification of relative neurite length (*n* = 3). K) Volcano plot showing gene expression changes associated with RU@PEI‐ZIF treatment relative to dsDNA‐ and senescence‐induced transcriptional states. L) Enrichment analyses of downregulated (left) and upregulated (right) pathways associated with RU@PEI‐ZIF treatment. M) GSEA showing suppression of SASP and neuron migration pathways in dsDNA‐treated NP cells versus RU@PEI‐ZIF‐treated NP cells, and suppression of cellular senescence and neurotrophic signaling pathways in senescent NP cells versus RU@PEI‐ZIF‐treated NP cells. N) Heatmap of representative senescence/SASP‐related genes across dsDNA, SEN, and RU@PEI‐ZIF groups. O) Heatmap of representative neuron migration‐associated genes across dsDNA, SEN, and RU@PEI‐ZIF groups. ^*^ *p* < 0.05, ^**^ *p* < 0.01, ^***^ *p* < 0.001, ^****^ *p* < 0.0001.

We next performed RNA sequencing of dsDNA‐treated NP cells, senescent NP cells, and RU@PEI‐ZIF‐treated senescent NP cells. Volcano plot analysis revealed widespread differential gene expression, identifying a subset of transcripts that were consistently dysregulated in both dsDNA‐treated and senescent cells but were partially restored following RU@PEI‐ZIF treatment. Notably, genes associated with the SASP and degenerative processes, including *Il1b, Cxcl2, Cdkn1a, Cdkn2a, Ntn1* and *Mmp3*, showed reduced expression, while those related to ECM homeostasis and adaptive stress responses, such as *Acan, Eln, Hmox1* and *Nqo1*, were upregulated (Figure 7K). Consistently, pathway enrichment analysis showed reduced enrichment of inflammatory, senescence‐associated, and pro‐innervation programs after RU@PEI‐ZIF treatment, including cellular response to interleukin‐1, TNF signaling, and cGAS–STING signaling, together with increased enrichment of programs related to ECM organization and DNA double‐strand break repair (Figure 7L). GSEA further supported this phenotypic reprogramming, revealing negative enrichment of transcriptional programs related to the SASP, neuronal migration, cellular senescence, and neurotrophin signaling (Figure 7M), as well as cytosolic DNA sensing and the cGAS–STING axis (Figure S11C). Finally, heatmap analysis further showed coordinated downregulation of genes within the senescence/SASP and neuronal migration networks (Figure 7N,O), together with reduced expression of representative DNA‐sensing inflammatory effectors (Figure S11D). Together, these transcriptomic, molecular, and functional findings indicated that RU@PEI‐ZIF suppressed cGAS–STING signaling and Netrin‐1‐associated pro‐innervation activity, supporting its subsequent in vivo evaluation.

### 2.7 RU@PEI‐ZIF Mitigates Disc Degeneration by Suppressing NP Senescence and Pain Hypersensitivity

The therapeutic effects of the nanoplatforms were assessed via intradiscal injection in a rat model of disc degeneration (Figure 8A). Before assessing therapeutic outcomes, we examined the local retention of Cy5.5‐labeled formulations by in vivo fluorescence imaging. Local fluorescence gradually declined but remained detectable at day 7, with PEI‐ZIF and RU@PEI‐ZIF generally showing stronger retention than RU@ZIF (Figure S12A,B), consistent with improved local retention after PEI modification. At the study endpoint, histological analysis of major organs (heart, liver, spleen, lungs, and kidneys) was performed with H&E staining. Tissue architecture was intact, and no inflammation, necrosis, edema, or structural abnormalities were observed (Figure S9E). Together, these observations supported short‐term tissue compatibility after intradiscal administration.

**FIGURE 8:**
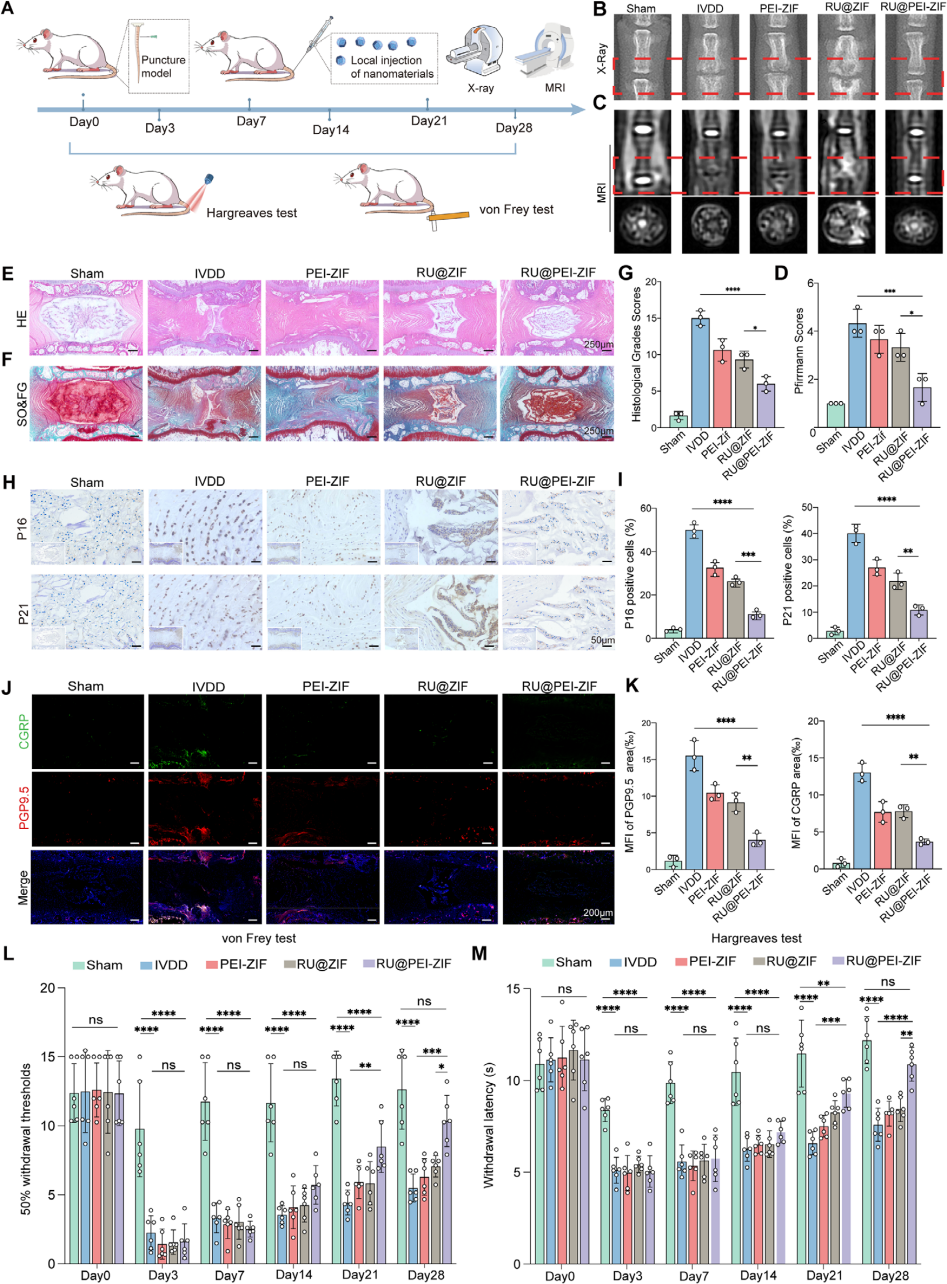
RU@PEI‐ZIF alleviates senescence‐driven pathological sensory innervation and discogenic pain. A) Schematic illustration of the rat caudal disc needle‐puncture model, intradiscal treatment regimen, and outcome assessments, including longitudinal behavioral testing by von Frey and Hargreaves tests, and radiological evaluation by X‐ray and MRI. B, C) Representative X‐ray (B) and T2‐weighted MRI (C) images of caudal intervertebral discs across the indicated groups. D) Pfirrmann scores based on T2‐weighted MRI assessment (*n* = 3). E, F) Representative H&E staining (E) and SO&FG staining (F) of intervertebral discs across the indicated groups. G) Histopathological degeneration scores of intervertebral discs across the indicated groups (*n* = 3). H, I) Representative immunohistochemical staining and quantification of the senescence markers P16 and P21 in disc sections across the indicated groups (*n =* 3). J, K) Immunofluorescence imaging and corresponding quantification of CGRP and PGP9.5 in disc sections across the indicated groups (*n* = 3). L) Mechanical sensitivity was assessed using the von Frey test, with 50% tail withdrawal thresholds measured in different experimental groups (*n* = 6). M) Thermal hyperalgesia was evaluated via the Hargreaves test in response to heat stimulation in different groups (*n* = 6). ns, not significant; ^*^ *p* < 0.05, ^**^ *p* < 0.01, ^***^ *p* < 0.001, ^****^ *p* < 0.0001.

We then assessed disc integrity by X‐ray and T2‐weighted MRI. The IVDD group showed disc‐space narrowing and loss of structural definition, whereas RU@PEI‐ZIF best preserved the disc space among treatment groups (Figure 8B). Consistently, RU@PEI‐ZIF‐treated discs retained stronger T2 signals than untreated IVDD discs, indicating better preservation of NP hydration and tissue structure (Figure 8C). Modified Pfirrmann scores were lower after RU@PEI‐ZIF treatment than in the IVDD group (Figure 8D). Although PEI‐ZIF and RU@ZIF produced partial improvements, RU@PEI‐ZIF yielded the lowest mean score among the treatment groups.

Histological analyses were consistent with the imaging findings. H&E staining showed NP shrinkage, a poorly defined NP–AF boundary, and extensive fibrotic remodeling in IVDD discs. RU@PEI‐ZIF preserved NP architecture and a clearer NP‐AF interface than the untreated IVDD group (Figure 8E). SO&FG staining showed severe proteoglycan depletion in the degeneration, PEI‐ZIF, and RU@ZIF‐treated groups, while RU@PEI‐ZIF markedly attenuated ECM loss (Figure 8F). Consistent with these features, RU@PEI‐ZIF produced the lowest histopathological degeneration score among the treatment groups (Figure 8G).

Next, we investigated whether structural preservation correlated with reduced NP cell senescence and aberrant sensory innervation. Immunohistochemistry showed increased P16‐ and P21‐positive cells in IVDD discs, whereas RU@PEI‐ZIF produced the largest reduction across the treatment groups (Figure 8H,I). Immunofluorescence similarly revealed pronounced accumulation of CGRP^+^ and PGP9.5^+^ sensory fibers in degenerative discs; RU@PEI‐ZIF reduced this accumulation significantly more than either component alone (Figure 8J,K). Longitudinal behavioral testing showed little separation among IVDD and treatment groups at the early time points. At later time points, RU@PEI‐ZIF increased mechanical withdrawal thresholds and prolonged thermal withdrawal latencies compared with the IVDD group (Figure 8L,M). Overall, these findings demonstrate that RU@PEI‐ZIF preserves disc structure, diminishes NP cell senescence, inhibits pathological sensory innervation, and alleviates discogenic pain.

### 2.8 RU@PEI‐ZIF Attenuates Degenerative Disc Pathology and Suppresses the dsDNA–cGAS–STING Axis

To determine whether RU@PEI‐ZIF altered cytosolic DNA sensing in vivo, we examined DNA damage, dsDNA burden, and cGAS–STING pathway‐associated signals in puncture‐induced IVDD discs. Immunofluorescence analysis showed strong γH_2_A signals in degenerative discs, indicating pronounced DNA damage. Treatment with PEI‐ZIF or RU@ZIF partially reduced γH_2_A signals, whereas the greatest reduction was observed in the RU@PEI‐ZIF group (Figure 9A). Consistently, degenerative discs exhibited marked dsDNA accumulation and elevated cGAS expression. PEI‐ZIF and RU@ZIF partially decreased these signals, whereas RU@PEI‐ZIF yielded the lowest mean dsDNA and cGAS signals among the treatment groups (Figure 9B,C). STING immunofluorescence showed a similar pattern, with the lowest signal observed in the RU@PEI‐ZIF group (Figure 9D,E). These findings indicate that RU@PEI‐ZIF attenuated dsDNA accumulation and cGAS–STING pathway‐associated signals in vivo.

**FIGURE 9:**
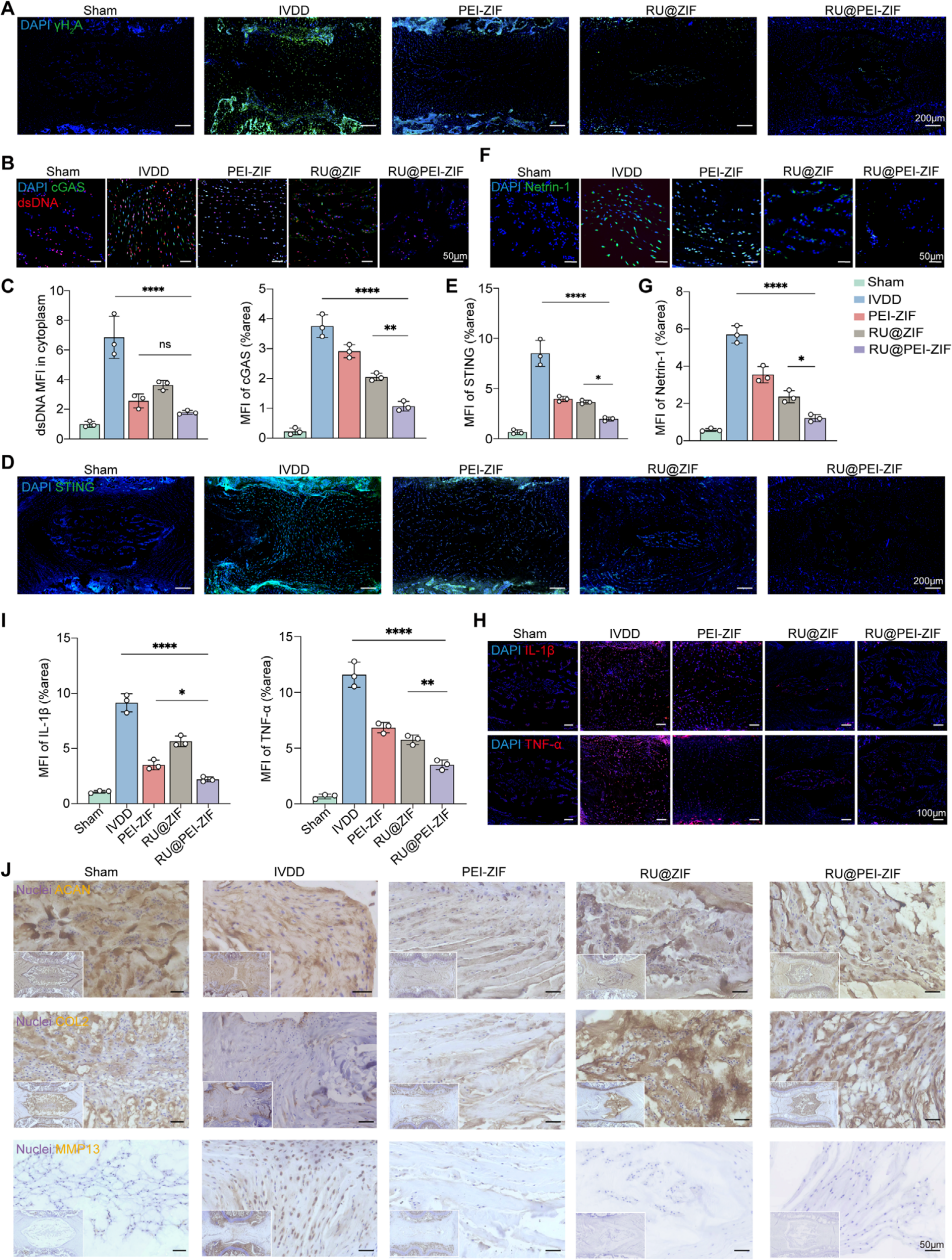
RU@PEI‐ZIF suppresses dsDNA–cGAS–STING signaling and remodels the degenerative disc microenvironment in vivo. A) Immunofluorescence imaging of γH_2_A in disc sections across the indicated groups. B, C) Immunofluorescence imaging and corresponding quantification of dsDNA and cGAS in disc sections across the indicated groups (*n* = 3). D, E) Immunofluorescence imaging and corresponding quantification of STING across the indicated groups (*n* = 3). F, G) Immunofluorescence imaging and corresponding quantification of Netrin‐1 across the indicated groups (*n* = 3). H, I) Immunofluorescence imaging and corresponding quantification of IL‐1β and TNF‐α across the indicated groups (*n* = 3). J) Representative immunohistochemical staining of ECM anabolic markers, including aggrecan and type II collagen, and the matrix catabolic enzyme MMP13 across the indicated groups. ns, not significant; ^*^ *p* < 0.05, ^**^ *p* < 0.01, ^****^ *p* < 0.0001.

We next examined whether these changes were accompanied by reduced inflammatory and neurotrophic signaling in the disc. Immunofluorescence analysis showed pronounced upregulation of Netrin‐1, IL‐1β, and TNF‐α in untreated degenerative discs. Treatment with RU@PEI‐ZIF reduced these inflammatory and neurotrophic signals more effectively than either comparator formulation (Figure 9F–I). Immunohistochemical analysis revealed that RU@PEI‐ZIF restored ECM balance by increasing anabolic proteins, including aggrecan and type II collagen, while reducing the catabolic enzyme MMP13 (Figure 9J).

Collectively, these findings indicate that RU@PEI‐ZIF reduces dsDNA accumulation and cGAS–STING‐associated signaling in degenerative discs, accompanied by attenuation of inflammatory and neurotrophic signals and improved ECM homeostasis. This coordinated remodeling of the disc microenvironment was consistent with preserved tissue architecture and reduced pain hypersensitivity. These findings further support the therapeutic potential of RU@PEI‐ZIF as a dual‐functional strategy for modulating the dsDNA–cGAS–STING axis in vivo.

## 3 Discussion

In this study, we identified dsDNA accumulation as a prominent feature of senescent NP cells and degenerative intervertebral discs. This accumulation was associated with cGAS–STING activation, increased Netrin‐1 expression, and an innervation‐permissive microenvironment. These findings provided the rationale for developing RU@PEI‐ZIF, a dual‐functional nanoplatform that combines PEI‐mediated dsDNA scavenging with local delivery of the cGAS inhibitor RU.521. In cellular and rat models, RU@PEI‐ZIF attenuated cellular senescence, inflammatory responses, and matrix catabolism. It also attenuated cGAS–STING signaling, Netrin‐1 expression, and pathological innervation while improving pain‐related outcomes. Collectively, these findings support the therapeutic potential of RU@PEI‐ZIF as a dual‐functional platform that combines dsDNA scavenging with cGAS inhibition to mitigate disc degeneration and pathological innervation.

Our findings indicate that dsDNA accumulation is not merely a passive by‐product of cellular damage but may act as an amplifier of NP cell senescence and a contributor to the pro‐innervation microenvironment associated with disc degeneration. cGAS recognizes cytosolic dsDNA and activates STING‐dependent inflammatory signaling, thereby linking cytosolic dsDNA accumulation to sterile inflammation and cellular senescence [33]. Consistent with this mechanism, exogenous dsDNA aggravated NP cell senescence and matrix catabolism, whereas extracellular dsDNA degradation by DNase I partially reversed these changes. Netrin‐1 is a secreted axon guidance cue that regulates axonal attraction and repulsion. Previous studies have shown that NP cell‐derived Netrin‐1 promotes axon growth and nerve innervation, whereas osteoclast‐derived Netrin‐1 mediates sensory innervation and spinal hypersensitivity [34]. In our study, pharmacological inhibition of cGAS reduced Netrin‐1 expression. Furthermore, knockdown of cGAS or Netrin‐1 diminished PC12 cell migration and neurite outgrowth. Together, these findings support a functional link between dsDNA‐responsive cGAS–STING signaling, Netrin‐1 induction, and pathological sensory innervation in disc degeneration, and provide a focused framework for further defining the NP‐cell‐specific connection between cGAS–STING activation and Netrin‐1 induction.

Guided by the key roles of dsDNA accumulation and cGAS activation in NP cell senescence, we sought a material platform capable of targeting this upstream pathway. Cationic polymers neutralize nucleic acids through electrostatic interactions, but their dose‐dependent cytotoxicity limits clinical application [35, 36]. To mitigate this limitation, we incorporated PEI at the ZIF‐8 nanoparticle interface, generating a formulation that retained dsDNA‐binding activity at a cytocompatible concentration while delivering RU.521 for local cGAS inhibition. At the tested concentration, RU@PEI‐ZIF retained dsDNA‐binding activity while remaining cytocompatible. The high loading capacity and tunable architecture of ZIF‐8 support sustained drug delivery, whereas its acid‐labile coordination bonds enable pH‐responsive release [37, 38]. Consistent with these properties, RU.521 release was enhanced under mildly acidic conditions. Following intradiscal administration, this pH‐responsive behavior may favor localized drug release within the degenerative disc [39]. Although degradation and biosafety were evaluated during the experimental period, the long‐term degradation, in vivo clearance, and safety of RU@PEI‐ZIF remain to be established. Further large‐animal studies and clinical trials are needed to support translation [40, 41].

The synergistic integration of dsDNA capture and cGAS inhibition underpins the therapeutic efficacy of RU@PEI‐ZIF in alleviating discogenic pain. By reducing the dsDNA burden and suppressing cGAS–STING signaling, RU@PEI‐ZIF produced the greatest reductions in SASP factors and Netrin‐1 release among the tested formulations. Although our analyses did not distinguish nuclear from mitochondrial dsDNA, nonspecific dsDNA sequestration by PEI combined with downstream cGAS inhibition by RU.521 may render the therapeutic effects of RU@PEI‐ZIF less dependent on DNA source. Nevertheless, the source‐specific contributions of dsDNA warrant further investigation [42]. In vivo, this coordinated intervention attenuated disc degeneration, pathological sensory innervation, and pain hypersensitivity. It also differs mechanistically from single‐modality approaches such as senolytic drugs, NSAIDs, and corticosteroids [43]. The increased cGAS and Netrin‐1 signals observed in human degenerative discs support the relevance of this pathway to human disease, although the limited sample size and clinical heterogeneity may restrict the generalizability of the human‐tissue findings. Overall, our work provides a mechanism‐guided strategy for targeting the dsDNA–cGAS axis to address both disc degeneration and discogenic pain.

## 4 Conclusion

In this study, we found that dsDNA accumulation contributes to NP cell senescence. Our data further support a functional role for dsDNA‐responsive cGAS–STING signaling and Netrin‐1 induction in the pro‐innervation phenotype of senescent NP cells. Guided by these findings, we developed RU@PEI‐ZIF, which combines dsDNA scavenging with cGAS inhibition in a single nanoplatform. In vitro, RU@PEI‐ZIF attenuated NP cell senescence and inflammatory, matrix‐catabolic, and pro‐innervation responses; in a rat model of IVDD, it reduced disc degeneration, pathological innervation, and pain hypersensitivity. Together, these findings provide a rationale for concurrently targeting pathological dsDNA accumulation and cGAS‐mediated DNA sensing to mitigate disc degeneration and pathological innervation.

## 5 Methods and Materials

### 5.1 Materials and Reagents

2‐methylimidazole, zinc acetate dihydrate (Zn(CH_3_COO)_2_·2H_2_O), and polyethyleneimine (PEI) were obtained from Aladdin (Shanghai, China); Deoxyribonucleic acid, cell counting kit‐8, DNase I and type II collagenase were all obtained from Sigma (St. Louis, MO, USA); Cy5.5 NHS ester and RU.521 were obtained from APExBIO (USA); BCA protein assay kit, hematoxylin‐Eosin (H&E) stain kit, Safranin O and Fast Green stain kit, Calcein‐AM/PI, phalloidin, DAB substrate kit, bovine serum albumin, and PicoGreen dsDNA quantification kit were all obtained from Solarbio (Beijing, China); ELISA kits for rat dsDNA, IL‐1β, TNF‐α, IL‐6 and Netrin‐1 were purchased from Upingbio (China); DAPI dihydrochloride, senescence β‐galactosidase staining kit, western blot reagents were all obtained from Beyotime Biotechnology (China); 4% paraformaldehyde was obtained from Servicebio (Wuhan, China); TRIzol reagent, Transwell insert, Lipofectamine 3000, Lipofectamine RNAiMAX Reagent and PBS were obtained from Thermo Fisher Scientific Inc. (USA).

### 5.2 Patients and Samples

Disc degeneration was evaluated by preoperative MRI, and the severity of each disc was assigned a Pfirrmann grade. Discs scoring I or II were treated as non‐degenerative controls, while those scoring III to V were identified as degenerative. All participants provided written informed consent. Inclusion criteria were as follows: clinical history and imaging findings consistent with the surgical diagnosis, and consent for sample collection and study participation. Exclusion criteria included spinal infection, tumor, autoimmune disease, metabolic bone disease, previous surgery at the sampled level, or severe systemic contraindications to surgery. The clinical characteristics of the patients are summarized in Table S1.

### 5.3 Single‐Cell Transcriptomic Analysis

Analysis of scRNA‐seq data from five public datasets (GSE165722, GSE199866, GSE222182, GSE233666, and GSE244889) deposited in the Gene Expression Omnibus (GEO) database was performed. Raw count matrices were processed in R using Seurat (version 5.0.1) [44]. Ambient RNA contamination and doublets were removed using DecontX [45] and DoubletFinder [46], respectively, followed by dataset integration with Harmony for batch correction. Dimensionality reduction and visualization were achieved using t‐distributed stochastic neighbor embedding (t‐SNE). NP cells were extracted for subclustering and downstream analysis. Senescence module scores were computed with the AddModuleScore function, utilizing the SenMayo gene set from MSigDB. Differentially expressed genes were identified using FindMarkers and analyzed for GO and KEGG enrichment, while CellChat (v1.6.1) assessed changes in intercellular communication related to NP cell senescence [47].

### 5.4 mRNA Sequencing and Analysis

Total RNA from NP cells was extracted using TRIzol and assessed for quality and concentration using a NanoDrop spectrophotometer and Agilent 2100 Bioanalyzer. mRNA was enriched using poly‐T oligo‐conjugated magnetic beads, fragmented, and reverse‐transcribed to synthesize cDNA. Libraries were prepared following standard protocols and sequenced on an Illumina platform. Raw reads were filtered to remove adapters and low‐quality sequences. The resulting clean reads were aligned to the Rattus norvegicus reference genome (mRatBn7.2) using STAR [48]. Gene‐level counts were generated using STAR. For downstream visualization, potential batch effects were assessed and corrected using the removeBatchEffect function in the limma package. Differential gene expression was assessed with DESeq2 (v1.38.3) [49]. Genes with adjusted p < 0.05 and |log_2_ fold change| > 1 were defined as differentially expressed. Enrichment of biological functions and pathways was analyzed using clusterProfiler (v4.6.0) [50].

### 5.5 Preparation of RU@ZIF, PEI‐ZIF, and RU@PEI‐ZIF Nanoparticles

2‐Methylimidazole (1.50 g), PEI (150 mg), and RU.521 (50 mg) were dissolved together in 75 mL of methanol. Zinc acetate dihydrate (0.50 g) was dissolved in 75 mL of methanol. The zinc solution was rapidly added to the ligand mixture under continuous stirring, and the reaction was maintained at 650 rpm for 2 h at room temperature. After the reaction, the nanoparticles were centrifuged and washed 3 times with anhydrous methanol. The particles were then dried in a vacuum oven at 60°C for 24 h to obtain the final RU@PEI‐ZIF formulation. RU@ZIF was prepared using the same protocol without PEI, whereas PEI‐ZIF was prepared without RU.521. Bare ZIF‐8 was prepared using the same procedure without PEI or RU.521.

### 5.6 Determination of Drug‐Loading Content and Encapsulation Efficiency

The drug‐loading content (DLC) and encapsulation efficiency (EE) of RU@ZIF and RU@PEI‐ZIF were determined using an indirect method. After nanoparticle preparation, the suspensions were centrifuged, and the supernatants and washing fractions were collected. Unencapsulated RU.521 was quantified by UV–vis spectrophotometry at 290 nm using a standard calibration curve, and the amount of loaded RU.521 was calculated by subtracting the unencapsulated amount from the initial amount added. The recovered nanoparticles were dried and weighed. DLC and EE were calculated as follows: DLC (%) = mass of loaded RU.521 / mass of drug‐loaded nanoparticles × 100%; EE (%) = mass of loaded RU.521 / initial mass of RU.521 × 100%.

### 5.7 Nanoparticle Characterization

The hydrodynamic diameter and zeta potential of the nanoparticles were characterized by DLS (Zetasizer Nano ZS90, Malvern Panalytical). XRD (Bruker D8 Venture, Germany) and FT‐IR (Nicolet iS5, Thermo Fisher Scientific, USA) were applied to analyze the crystal structure and chemical composition, respectively. Morphological properties were measured using FE‐SEM (TESCAN MIRA LMS) and FE‐TEM (JEOL JEM‐F200). Energy‐dispersive X‐ray spectroscopy (EDS) was used for elemental composition and mapping. TGA (TA TGA55, USA) was used for thermogravimetric analysis under a nitrogen atmosphere. The specific surface area was determined using BET analysis (Micromeritics ASAP 2460, USA).

### 5.8 Drug Release Profile

An amount of RU@PEI‐ZIF nanoparticles containing 5 µg of loaded RU.521, as calculated from the experimentally determined drug‐loading content, was suspended in 1 mL of PBS at pH 7.4 or 6.0 at room temperature. At 24, 48, 72, and 96 h, the suspensions were centrifuged to collect the supernatant. The concentration of RU.521 in each supernatant was determined by UV–vis spectrophotometry at 290 nm against a standard curve. RU.521 release (%) = released RU.521 / initially loaded RU.521 × 100%.

### 5.9 dsDNA Binding Capacity

To quantify dsDNA‐binding capacity, 1 mg of RU@ZIF or RU@PEI‐ZIF nanoparticles were incubated with 5–30 µg of calf thymus DNA under gentle agitation at room temperature for 4 h. Following incubation, nanoparticles were removed by centrifugation, and the remaining unbound DNA in the supernatant was quantified using the Quant‐iT PicoGreen dsDNA Assay Kit. The amount of bound DNA was calculated by subtracting residual unbound DNA from the initial input.

### 5.10 Isolation, Culture, and Treatment of Cells

NP tissue from the tail intervertebral discs of Sprague‐Dawley rats (4 weeks old) was harvested, finely minced, and incubated with 2% type II collagenase at 37°C for 4 h. The suspension was centrifuged at 1000 × g for 5 min. The resulting pellet was resuspended and cultured in complete medium (DMEM/F12, Gibco, USA) supplemented with 10% fetal bovine serum (FBS, Gibco, USA) and 1% penicillin‐streptomycin at 37 °C under 5% CO_2_. Passage took place at 80% confluence, with Passage 2 (P2) cells as the control and Passage 8 (P8) cells for replicative senescence modeling.

PC‐12 (low differentiation) cells (RRID: CVCL_0481) were kindly provided by Wuhan Procell Life Science & Technology Co., Ltd. (China). These cells were cultured in RPMI‐1640 medium (Gibco, USA) supplemented with 10% FBS and 1% penicillin‐streptomycin at 37°C in a 5% CO_2_ humidified incubator. For migration and non‐contact co‐culture assays, PC12 cells were transferred to serum‐free RPMI‐1640 medium before seeding, as described in the corresponding experimental procedures.

### 5.11 H_2_O_2_‐Induced Oxidative Stress and DNase I Treatment

NP cells were exposed to 100 µm H_2_O_2_ for 2 h to establish an oxidative stress‐induced injury model. The H_2_O_2_‐containing medium was then removed, and the cells were washed with PBS and cultured in fresh complete medium. For extracellular dsDNA clearance, senescent NP cells or H_2_O_2_‐treated NP cells were subsequently treated with DNase I at a final concentration of 50 U mL^−1^.

### 5.12 dsDNA Stimulation and Pharmacological Inhibition

To induce dsDNA‐driven pathological responses, NP cells were transfected with calf thymus DNA (10 µg mL^−1^) using Lipofectamine 3000 for 2 h. Cells were subsequently treated with the cGAS inhibitor RU.521 (10 µg mL^−1^) for an additional 2 h, unless otherwise specified.

### 5.13 Cell Transfection

Small interfering RNAs (siRNAs; sequences in Table S2) were obtained from Tenuo (Guangzhou, China). NP cells were transfected using Lipofectamine RNAiMAX according to the manufacturer's instructions. Knockdown efficiency was evaluated by a western blot assay.

### 5.14 Transwell Migration and Non‐Contact Co‐Culture Assays

Migration of PC12 cells was assessed using 24‐well Transwell chambers with 8.0 µm pores. NP cells, following treatment as specified, were seeded in the lower chamber with serum‐free DMEM/F12, while PC12 cells were seeded in the upper chamber with serum‐free RPMI‐1640 medium (200 µL per insert). After a 24‐h incubation, cells on the upper membrane were removed. The migrated cells on the lower membrane were stained with 0.1% crystal violet and counted across five fields using an inverted microscope.

NP cells were seeded in the upper chamber of 0.4 µm Transwell inserts for non‐contact co‐culture, and PC12 cells were seeded in the lower chamber. After 24 h, PC12 cells were fixed, permeabilized, blocked, and incubated overnight at 4°C with an anti‐β3‐tubulin primary antibody, followed by incubation with an Alexa Fluor 488‐conjugated secondary antibody for 1 h. Nuclei were counterstained with DAPI, and images were captured using confocal microscopy. Neurite length was quantified to assess neurite extension.

### 5.15 SA‐β‐Gal Staining

NP cells were grown in standard culture conditions and treated according to the treatment instructions. After treatment, cells were rinsed with PBS and fixed for 15 min at ambient temperature. The fixed cells were then incubated with SA‐β‐Gal staining solution at 37°C without CO_2_ for 24 h. Blue‐stained cells were examined under a light microscope, and the proportion of SA‐β‐Gal‐positive cells was determined from five randomly selected fields per well.

### 5.16 Cytocompatibility and Cell Morphology Assessment

NP cells were seeded at 5 × 10^4^ cells per well in 96‐well plates and allowed to adhere overnight under normal culture conditions. Cells were exposed to different concentrations of RU@ZIF, PEI‐ZIF, or RU@PEI‐ZIF for 24 h. Cell viability was assessed with CCK‐8 absorbance readings at 450 nm after 2 h of incubation. Live/dead staining with Calcein‐AM/PI was performed after nanoparticle treatment. Cytoskeletal organization and cell spreading were examined by staining with phalloidin and analyzed under fluorescence microscopy.

### 5.17 Immunofluorescence Staining

Cells were seeded on coverslips and treated as described. After washing cells with PBS, cells were fixed with 4% paraformaldehyde. Permeabilization was performed using 0.3% Triton X‐100 for 15 min. Samples were blocked with 10% goat serum for 1 h at 37°C. Primary antibodies were applied overnight at 4°C. After PBST washes, fluorophore‐conjugated secondary antibodies (Table S3) were applied, and nuclei were counterstained with DAPI. Images were captured with a confocal microscope.

### 5.18 ELISA

Conditioned media were collected from cells that had been treated as described and centrifuged at 3000 × g for 10 min to remove debris. The supernatants were collected for analysis. Levels of dsDNA, Netrin‐1, IL‐1β, IL‐6, and TNF‐α were measured using commercial ELISA kits according to the manufacturer's instructions.

### 5.19 Rat Disc Degeneration Model Construction

Eight‐week‐old male Sprague–Dawley rats were obtained from the Experimental Animal Center of Southern Medical University and housed under standard laboratory conditions with free access to food and water. All experimental procedures were approved by the Institutional Animal Ethics Committee. Rats were randomly assigned to five groups. Anesthesia was induced by intraperitoneal injection of sodium pentobarbital (45 mg kg^−1^). To induce disc degeneration, a 21G needle was inserted dorsally into the intervertebral disc, advanced through the center of the disc to the opposite side, rotated 360°, and maintained in position for 30 s. Sham‐operated rats underwent skin puncture without disc injury. One week after needle puncture, rats received intradiscal injections of PBS, RU@ZIF, PEI‐ZIF, or RU@PEI‐ZIF suspension (5 µL, 10 µg mL^−1^) using a 31G Hamilton syringe, with treatment administered once weekly. Mechanical withdrawal thresholds and thermal withdrawal latencies were assessed longitudinally before each treatment. At 4 weeks after needle puncture, the final behavioral assessment and radiological evaluation were performed, after which the rats were euthanized, and the caudal discs were collected for histological and molecular analyses.

### 5.20 In Vivo Fluorescence Imaging of Nanoparticle Retention

To evaluate the intradiscal retention of the different nanoparticle formulations, rats received intradiscal injections of 5 µL of Cy5.5‐labeled RU@ZIF, PEI‐ZIF, or RU@PEI‐ZIF, with an equivalent amount of Cy5.5 administered in each group. Fluorescence images were acquired on days 1, 3, and 7 after injection using an IVIS Spectrum imaging system (PerkinElmer, USA) with an excitation wavelength of 640 nm and a 680–700 nm emission filter for Cy5.5 detection.

### 5.21 Radiological Evaluation

At 4 weeks post‐puncture, rat tails were imaged using X‐ray radiography (Philips Digital Diagnost) and 3.0‐T MRI (Philips Intera Achieva). T2‐weighted images and T2 mapping images were acquired. Disc degeneration was graded in a blinded manner using the modified Pfirrmann grading system based on disc structure and signal intensity.

### 5.22 Mechanical Withdrawal Threshold and Hargreaves Tests

Mechanical sensitivity was assessed using von Frey filaments. Rats were placed individually in a three‐compartment enclosure with a wire‐mesh floor and allowed to habituate for 20 min. Filaments were applied to the ventral base of the tail, starting at 2 g, for up to 6 s. A positive response was defined as tail withdrawal, licking, or shaking. Each filament force was applied five times consecutively, with an interval of at least 2 min between stimuli. Withdrawal thresholds were determined using the up–down paradigm, in which a lighter filament was applied after a positive response and a heavier filament after no response, and were expressed as the 50% withdrawal thresholds. Thermal sensitivity was assessed using a Hargreaves‐type tail‐flick assay system (ZL‐031, Anhui Yaokun Biotechnology Co., Ltd., China). Rats were habituated in an acrylic chamber for 20 min and then exposed to a radiant infrared stimulus directed to the ventral surface of the tail near the punctured disc level at 50% maximal intensity. The latency to a withdrawal response, including tail withdrawal, licking, biting, or shaking, was recorded, with an automatic cut‐off time of 20 s to prevent tissue injury. Each animal underwent four trials per time point, separated by a minimum of 5 min, and the average latency was calculated for analysis.

### 5.23 Adeno‐Associated Virus (AAV)‐Mediated Knockdown In Vivo

Eight‐week‐old male Sprague–Dawley rats (∼200 g) were randomly assigned to receive either a control AAV vector (AAV‐null) or an AAV vector encoding NTN1 shRNA. After anesthesia with intraperitoneal pentobarbital sodium (45 mg kg^−1^), AAV vectors were delivered intradiscally into the disc using a 31G Hamilton microsyringe (2 µL per disc; 1.5 × 10^13^ vg mL^−1^). The needle tract was immediately sealed with bone wax to minimize leakage. Animals were allowed to recover for 7 days before disc degeneration. AAV6 vectors were obtained from Guangzhou Tenuo Biotechnology Co., Ltd. (Guangzhou, China). Seven days after AAV injection, needle‐puncture‐induced disc degeneration was performed as described above. Rats were euthanized 4 weeks after puncture for subsequent analysis.

### 5.24 Western Blotting

NP cells or disc tissues were lysed in RIPA buffer containing protease and phosphatase inhibitors. Protein content was quantified using a BCA assay. Equal amounts of protein were separated on 12% SDS–PAGE gels and transferred to PVDF membranes (Millipore). Membranes were blocked with 5% non‐fat milk and then incubated with primary antibodies at 4 °C overnight. After washing with TBST, membranes were treated with HRP‐conjugated secondary antibodies (Table S3) for 2 h at room temperature. Signals were performed using enhanced chemiluminescence and captured with a Bio‐Rad ChemiDoc MP system.

### 5.25 Histological Evaluation, Immunohistochemistry, and Immunofluorescence

Rat disc tissues were collected after imaging and fixed in 4% paraformaldehyde for 1 week, followed by decalcification in 10% EDTA for 4 weeks. Human IVD samples were fixed directly in 4% paraformaldehyde. All tissues were embedded in paraffin and sectioned at 5–8 µm. Histological staining was performed following the manufacturer's protocols. Sections were examined under a light microscope, and grading was performed in a blinded manner. Immunohistochemistry was conducted using standard procedures. After deparaffinization, rehydration, and antigen retrieval, sections were blocked and incubated with primary antibodies overnight at 4 °C. Sections were then incubated with appropriate secondary antibodies (Table S3). Signals were visualized using DAB substrate, followed by hematoxylin counterstaining. Representative images were captured for analysis.

### 5.26 Statistical Analysis

All experiments were performed with at least three biological replicates. Data are presented as mean ± standard deviation (s.d.). Differences between two groups were analyzed using a two‐tailed unpaired Student's *t*‐test. For comparisons involving more than two groups, one‐way ANOVA followed by Tukey's multiple‐comparison test was applied. Longitudinal behavioral data were analyzed using two‐way ANOVA followed by Tukey's multiple‐comparison test. Statistical analyses were conducted using SPSS software, and *p* < 0.05 was considered statistically significant.

### 5.27 Ethics Statement

All procedures involving human specimens were reviewed and approved by the Ethics Committee of the Third Affiliated Hospital of Southern Medical University (Approval No. 2025‐ER‐139). Written informed consent was obtained from all participants before collection of human NP tissues. All animal experiments were performed in accordance with protocols approved by the Animal Ethics Committee of Southern Medical University, Guangdong, China (Approval No. NYSY‐LAEC‐25003).

## Author Contributions

Q.L., C.L., and L.Z. conceptualized the study and provided overall supervision. Z.H., T.C., Z.L., R.J., and B.X. designed and executed the experiments. Z.H., X.H., Y.L., and K.Z. analyzed and interpreted the data. Z.H., T.C., and Z.L. composed the manuscript. Z.H., T.C., Z.L., X.H., R.J., Y.L., B.X., Q.L., C.L., H.Y. and L.Z. participated in the manuscript review. All authors reviewed and approved the final version of the paper.

## Funding

This work was supported by the National Natural Science Foundation of China (82402798; 82472484; 32471431; 82572825), the Natural Science Foundation of Guangdong Province (2025A1515011844), the Guangdong Basic and Applied Basic Research Foundation (2025A1515011845), the Guangzhou Science and Technology Plan Project of China (2025A04J3679), and the President Funding of Third Affiliated Hospital of Southern Medical University (YQ202309).

## Conflicts of Interest

The authors declare no conflicts of interest.

## Supporting information




**Supporting File**: advs78215‐sup‐0001‐SuppMat.docx.

## Data Availability

The data that support the findings of this study are available from the corresponding author upon reasonable request.
