# Supplementary material for: A Dual‐Functional Nanoplatform Targeting the dsDNA–cGAS Axis to Mitigate Disc Degeneration and Aberrant Innervation
Source: Adv Sci (Weinh). 2026 Oct 12:e78215. Online ahead of print. doi: 10.1002/advs.78215 (PMC13658893; doi:10.1002/advs.78215)
Supplement: Supplementary file 1 [file ADVS-9999-e78215-s001.docx]

Supporting Information

A dual-functional nanoplatform targeting the dsDNA–cGAS axis to mitigate disc degeneration and aberrant innervation

Zhaojun He, Tianyu Chen, Zezheng Liu, Xinlin Huang, Ruitangbin Jiang, Yihao Liu, Baojing Xu, Yuxiang Li, Kangning Zhao, Huibo Yan, Lei Zhou*, Can Liu*, Qingchu Li*

Z. He, T. Chen, Z. Liu, X. Huang, R. Jiang, Y. Liu, B. Xu, Y. Li, K. Zhao, H. Yan, C. Liu, Q. Li

Department of Orthopedics, Orthopedic Hospital of Guangdong Province, Academy of Orthopedics Guangdong Province, The Third Affiliated Hospital of Southern Medical University, Guangzhou 510560, China
E-mail: liucanspine@smu.edu.cn (C. Liu); lqc16@263.net (Q. Li)

L. Zhou

Department of Orthopaedic Surgery, Guangzhou Key Laboratory of Spine Disease Prevention and Treatment, The Third Affiliated Hospital, Guangzhou Medical University, Guangzhou, 510560, China

E-mail: zhoul@gzhmu.edu.cn (L. Zhou)

**Supplementary Figures and Tables Legends**

**Figure S1.** Human degenerative disc tissues exhibit elevated senescence, matrix remodeling, and cGAS/Netrin-1 expression. A) Representative MRI scans of human discs from control and IVDD groups. B) Representative immunohistochemical staining and quantification of P16 and P21 in human disc tissues from control and IVDD groups (*n*=3). C) Representative immunohistochemical staining and quantification of MMP13 and aggrecan in human disc tissues from control and IVDD groups (*n*=3). D) Representative immunohistochemical staining and quantification of cGAS and Netrin-1 in human disc tissues from control and IVDD groups (*n*=3). ***P* < 0.01, ****P* < 0.001, *****P* < 0.0001.

**Figure S2.** IVDD is associated with increased expression of DNA damage, senescence, inflammatory, and sensory innervation markers. A) Quantification of the Western blot analyses shown in Figure 2 for γH_2_A, P16, P21, PGP9.5, and CGRP in control (Con) and IVDD disc tissues (*n*=3). B) Quantification of the Western blot analyses shown in Figure 2 for IL-1β, TNF-α, IL-6, and MMP13 (*n*=3). ***P* < 0.01, ****P* < 0.001, *****P* < 0.0001.

**Figure S3.** Elevated DNA damage, inflammation, matrix catabolism, and angiogenesis in degenerative disc tissues. A) Immunofluorescence imaging and corresponding quantification of γH_2_A, IL-1β, TNF-α, and MMP13 in disc tissues from control and IVDD groups (*n*=3). B) Immunofluorescence imaging and corresponding quantification of CD31 in disc tissues from control and IVDD groups (*n*=3). ****P* < 0.001, *****P* < 0.0001.

**Figure S4.** H_2_O_2_ induces DNA damage-associated and pro-innervation signaling in NP cells. A) Volcano plot showing differentially expressed genes in H_2_O_2_-treated NP cells relative to Control NP cells. B) Functional enrichment analysis of the upregulated pathways in H_2_O_2_-treated NP cells. C, D) GSEA showing enrichment of positive regulation of neuron projection development (C) and double-strand break repair (D) in H_2_O_2_-treated NP cells relative to Control NP cells. E) Representative immunofluorescence images and corresponding quantification of cGAS, STING, and Netrin-1 expression in Control and H_2_O_2_-treated NP cells (*n*=3). ***P* < 0.01, ****P* < 0.001, *****P* < 0.0001.

**Figure S5.** Extracellular dsDNA promotes DNA damage, cellular senescence, and inflammatory responses in NP cells. A) Quantification of the Western blot analyses shown in Figure 3 for γH_2_A, P16, and P21 in Control and dsDNA-treated NP cells (*n*=3). B) Quantification of the Western blot analyses shown in Figure 3 for IL-1β, TNF-α, IL-6, and MMP13 in Control and dsDNA-treated NP cells (*n*=3). ***P* < 0.01, ****P* < 0.001, *****P* < 0.0001.

**Figure S6.** DNase I-mediated extracellular dsDNA clearance attenuates NP cell senescence**.** A) Representative SA-β-Gal staining and quantification of the SA-β-Gal-positive cells in the indicated groups (*n*=3). B) Representative immunofluorescence images and corresponding quantification of P16, P21, MMP13, and ACAN expression in the indicated groups (*n*=3). **P* < 0.05, ***P* < 0.01, ****P* < 0.001.

**Figure S7.** cGAS knockdown attenuates dsDNA-induced NP cell-driven neuronal migration and neurite outgrowth. A) Transfection efficiency of sicGAS-GFP in NP cells. B) Western blot analysis and quantification of cGAS knockdown in NP cells transfected with a non-targeting control (siNC) or three independent siRNAs (*n*=3). C) Western blot analysis and quantification of STING, Netrin-1, IL-1β, and TNF-α in NP cells subjected to the indicated dsDNA and siRNA treatments (*n*=3). D) Representative images and quantification of PC12 migration and β3-tubulin-positive neurite outgrowth in response to conditioned media from the indicated NP cell groups (*n*=3). ns, not significant; **P* < 0.05, ***P* < 0.01, ****P* < 0.001, *****P* < 0.0001.

**Figure S8.** Netrin-1 knockdown attenuates senescent NP cell-driven neurite outgrowth and pathological sensory innervation. A) Transfection efficiency of siNTN1-GFP in NP cells. B) Western blot analysis and quantification of Netrin-1 knockdown in NP cells transfected with a non-targeting control (siNC) or three independent siRNAs (*n*=3). C) Representative images and quantification of Transwell migration assays evaluating PC12 cell migration in response to conditioned media from the indicated NP cell groups (*n*=3). D) Representative β3-tubulin immunofluorescence images and quantification of PC12 neurite outgrowth in response to conditioned media from the indicated NP cell groups (*n*=3). E) Immunofluorescence imaging and corresponding quantification of CGRP and PGP9.5 in degenerative discs treated with AAV-null or AAV-NTN1 shRNA (*n*=3). ***P* < 0.01, ****P* < 0.001, *****P* < 0.0001.

**Figure S9.** Cytocompatibility and biosafety evaluation of the nanoplatforms. A) Representative immunofluorescence images of NP cells incubated with Cy5.5-labeled nanoparticles (PEI-ZIF, RU@ZIF and RU@PEI-ZIF); single-channel Cy5.5 insets highlight intracellular nanoparticle distribution. B) Viability of NP cells after 24 h of treatment with PEI-ZIF, RU@ZIF and RU@PEI-ZIF across the indicated concentrations (*n*=3). C) Representative phalloidin staining showing NP cell adhesion and cytoskeletal organization following the indicated treatments. D) Representative live/dead staining of NP cells after nanoparticle exposure. E) Representative H&E staining of major organs, including the heart, liver, spleen, lung, and kidney, from the indicated treatment groups.

**Figure S10.** RU@PEI-ZIF suppresses cGAS–STING–Netrin-1 signaling and inflammatory responses in senescent NP cells. A) Quantification of the Western blot analyses shown in Figure 7 for cGAS, STING, Netrin-1, IL-1β, TNF-α, and IL-6 in the indicated groups (*n*=3). **P* < 0.05, ***P* < 0.01, ****P* < 0.001, *****P* < 0.0001.

**Figure S11.** RU@PEI-ZIF attenuates inflammatory cytokine expression and inhibits cytosolic DNA-sensing pathways in senescent NP cells. A) Immunofluorescence imaging and corresponding quantification of IL-1β across the indicated groups (*n*=3). B) Immunofluorescence imaging and corresponding quantification of TNF-α across the indicated groups (*n*=3). C) GSEA showing negative enrichment of the cytosolic DNA-sensing pathway and the cGAS–STING axis in dsDNA-treated NP cells versus RU@PEI-ZIF-treated NP cells (top), and in senescent NP cells versus RU@PEI-ZIF-treated NP cells (bottom). D) Heatmap of representative genes associated with cGAS–STING/cytosolic DNA-sensing signaling across dsDNA, SEN, and RU@PEI-ZIF groups. ***P* < 0.01, ****P* < 0.001, *****P* < 0.0001.

**Figure S12.** In vivo retention of Cy5.5-labeled nanoparticles in rat discs. A) Representative IVIS fluorescence images of Cy5.5-labeled PEI-ZIF, RU@ZIF, and RU@PEI-ZIF nanoparticles following intradiscal injection on days 1, 3, and 7. B) Quantitative analysis of the normalized radiant efficiency within the disc region of interest (*n*=3). The radiant efficiency on day 1 was normalized to 100%. **P* < 0.05.

**Table S1.** NP Sample information and demographics.

| Case No. | Age (years) | Gender | Pfirrmann grade |
| --- | --- | --- | --- |
| Case 1 | 42 | female | I |
| Case 2 | 21 | female | II |
| Case 3 | 27 | male | II |
| Case 4 | 36 | female | I |
| Case 5 | 32 | male | I |
| Case 6 | 57 | female | IV |
| Case 7 | 61 | female | IV |
| Case 8 | 42 | female | III |
| Case 9 | 55 | male | IV |
| Case 10 | 56 | male | III |

**Table S2.** Sequences of the siRNAs and shRNA.

| Gene | species | Forward (5’→3’) | Reverse (5’→3’) |
| --- | --- | --- | --- |
| siNtn1-1 | Rat | GGUAAGACCUGCAAUCAAA | UUUGAUUGCAGGUCUUACC |
| siNtn1-2 | Rat | GCAACUCUUCCGAUCCCAA | UUGGGAUCGGAAGAGUUGC |
| siNtn1-3 | Rat | GCUCCUGUCACCUCUGCAA | UUGCAGAGGUGACAGGAGC |
| shNtn1 | Rat | CGGCCTCAGTGAGCGA | AGGAACCCCTAGTGATG |
| sicGAS-1 | Rat | AUAACAUCAAAUUCAUUAGGA | CUAAUGAAUUUGAUGUUAUGU |
| sicGAS-2 | Rat | UUUCGUAAUAUUCUUCUAGCU | CUAGAAGAAUAUUACGAAACU |
| sicGAS-3 | Rat | AUUCACAACAUGUUUUUCCUA | GGAAAAACAUGUUGUGAAUCU |

**Table S3.** Antibodies used in this study.

| Product name | Catalog | Manufacturer | Dilution Ratio |
| --- | --- | --- | --- |
| Anti-P16^Ink4a^ | ab54210 | Abcam | 1:2000 (WB); 1:200 (IF); 1:100 (IHC) |
| Anti-P21^cilp^ | 14-6715-81 | Invitrogen | 1:1000 (WB); 1:200 (IF); 1:100 (IHC) |
| Anti-γH_2_A | ab81299 | Abcam | 1:2500 (WB); 1:200 (IF) |
| Anti-MMP13 | A11148 | Abclonal | 1:1000 (WB); 1:100 (IF); 1:100 (IHC) |
| Anti-Collagen II | sc-52658 | Santa Cruz | 1:50 (IHC) |
| Anti-Aggrecan | 13880-1-AP | Proteintech | 1:100 (IHC) |
| Anti-IL-1β | 66737-1-Ig | Proteintech | 1:200 (IF) |
| Anti-IL-1β | YP-Ab-16004 | Upingbio | 1:2500 (WB) |
| Anti-TNF-α | sc-52746 | Santa Cruz | 1:2000 (WB); 1:200 (IF) |
| Anti-IL-6 | sc-57315 | Santa Cruz | 1:2000 (WB) |
| Anti-CGRP | PA5-114931 | Invitrogen | 1:1000 (WB); 1:100 (IF) |
| Anti-PGP9.5 | ab108986 | Abcam | 1:1000 (WB); 1:100 (IF) |
| Anti-dsDNA | sc-58749 | Santa Cruz | 1:200 (IF) |
| Anti-cGAS | HA500023 | Huabio | 1:1000 (WB); 1:200 (IF) |
| Anti-STING | HA722832 | Huabio | 1:1000 (WB); 1:100 (IF) |
| Anti-Netrin-1 | ab126729 | Abcam | 1:2000 (WB); 1:200 (IF) |
| Anti-beta3-Tubulin | 5568 | Cell Signaling Technology | 1:200 (IF) |
| Anti-CD31 | ab64543 | Abcam | 1:200 (IF) |
| Goat anti-Rabbit IgG (H+L) Alexa Fluor™ 488 Conjugate | A-11008 | Invitrogen | 1:500 (IF) |
| Goat anti-Mouse IgG (H+L) Alexa Fluor™ 568 Conjugate | A-11004 | Invitrogen | 1:500 (IF) |
| Goat anti-Rabbit IgG (H+L) HRP Conjugate | 31460 | Invitrogen | 1:20000 (WB); 1:300 (IHC) |
| Goat anti-Mouse IgG (H+L) HRP Conjugate | 31430 | Invitrogen | 1:20000 (WB); 1:300 (IHC) |
